# Influence of Proteome Profiles and Intracellular Drug Exposure on Differences in CYP Activity in Donor-Matched Human Liver Microsomes and Hepatocytes

**DOI:** 10.1021/acs.molpharmaceut.1c00053

**Published:** 2021-03-19

**Authors:** Christine Wegler, Pär Matsson, Veronica Krogstad, Jozef Urdzik, Hege Christensen, Tommy B. Andersson, Per Artursson

**Affiliations:** † Department of Pharmacy, Uppsala University, 752 37 Uppsala, Sweden; ‡ DMPK, Research and Early Development Cardiovascular, Renal and Metabolism, BioPharmaceuticals R&D, AstraZeneca, 431 50 Gothenburg, Sweden; § Department of Pharmaceutical Biosciences, School of Pharmacy, University of Oslo, 0315 Oslo, Norway; ∥ Department of Surgical Sciences, Uppsala University, 751 85 Uppsala, Sweden; ⊥ Department of Pharmacy and Science for Life Laboratory, Uppsala University, 752 37 Uppsala, Sweden

**Keywords:** human liver hepatocytes, human liver microsomes, drug clearance, protein quantification, intracellular unbound drug concentration

## Abstract

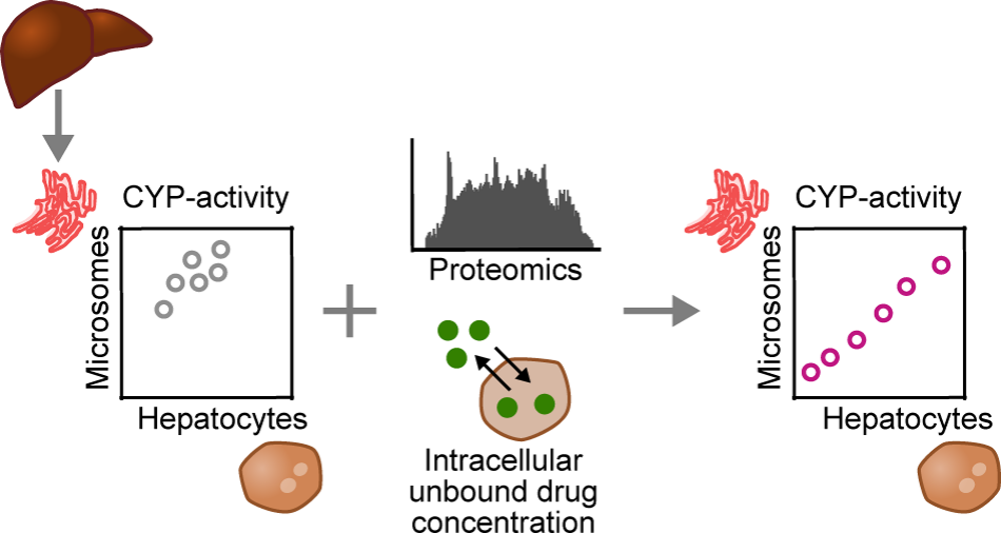

Human liver microsomes (HLM) and human hepatocytes (HH) are important *in vitro* systems for studies of intrinsic drug clearance (CL_int_) in the liver. However, the CL_int_ values are often in disagreement for these two systems. Here, we investigated these differences in a side-by-side comparison of drug metabolism in HLM and HH prepared from 15 matched donors. Protein expression and intracellular unbound drug concentration (Kp_uu_) effects on the CL_int_ were investigated for five prototypical probe substrates (bupropion–CYP2B6, diclofenac–CYP2C9, omeprazole–CYP2C19, bufuralol–CYP2D6, and midazolam–CYP3A4). The samples were donor-matched to compensate for inter-individual variability but still showed systematic differences in CL_int_. Global proteomics analysis outlined differences in HLM from HH and homogenates of human liver (HL), indicating variable enrichment of ER-localized cytochrome P450 (CYP) enzymes in the HLM preparation. This suggests that the HLM may not equally and accurately capture metabolic capacity for all CYPs. Scaling CL_int_ with CYP amounts and Kp_uu_ could only partly explain the discordance in absolute values of CL_int_ for the five substrates. Nevertheless, scaling with CYP amounts improved the agreement in rank order for the majority of the substrates. Other factors, such as contribution of additional enzymes and variability in the proportions of active and inactive CYP enzymes in HLM and HH, may have to be considered to avoid the use of empirical scaling factors for prediction of drug metabolism.

## Introduction

Intrinsic hepatic drug clearance influences drug bioavailability and exposure. To investigate this, *in vitro* models are often used during drug discovery and development. The two most commonly used models are isolated hepatocytes and liver microsomes.^1−9^ Isolated hepatocytes are the gold standard because these cells capture most of the factors influencing hepatic intrinsic clearance (CL_int_). They are used in various configurations to quantify metabolic activity as well as uptake and efflux transport of drugs and metabolites.^10^ However, liver microsomes are usually the first screening tool in studies of metabolic clearance because of their low cost and ease of access.^11^ Microsomes are derived by subcellular fractionation, with enrichment of the endoplasmic reticulum (ER).^12^ Many membrane-bound drug metabolizing enzymes are located in the ER, including cytochrome P450s (CYPs) and many UDP-glucuronosyltransferases (UGTs). Different results are often obtained from the hepatocytes and microsomes,^1,2,7,13,14^ but the reasons for these differences are not fully understood.

Mass spectrometry-based proteomics is increasingly used to investigate the protein content of the various drug metabolizing enzymes in microsomal preparations. We and others have shown that CYP and UGT enzymes are not enriched to the expected degree in subcellular fractions compared to the unfractionated homogenate.^15,16^ Large amounts of the ER-associated proteins are lost in the early fractionation steps,^15,17^ and the microsomes contain proteins from organelles other than the ER.^18,19^ Despite these studies on the protein composition of microsomes, no comprehensive analyses have compared the proteomes of liver microsomes, liver homogenates, and hepatocytes from the same donor. These analyses would show the relative impact of variability from the fractionation process and from inter-individual variation. Furthermore, the effect of the variable degree of microsomal protein enrichment on the microsomal metabolic activity has not been extensively investigated.

In contrast to microsomes, hepatocytes have an intact plasma membrane barrier that drug compounds must permeate in order to be metabolized by the intracellular phase I and II enzymes. For low-permeability drugs, the passage across the cell membrane can be rate limiting as active transport mechanisms can both facilitate and limit the cellular drug accumulation. The extent of drug metabolism in hepatocytes can be greatly influenced by the intracellular concentration of unbound drug, that is, how much drug is present inside the hepatocyte in accessible form.^20^ We recently observed that differences between biochemical and cellular potency assays could be bridged by taking into account the intracellular unbound drug concentration (expressed as intracellular bioavailability).^21^ We also found that over-prediction of time-dependent CYP inhibition could be resolved by incorporating the intracellular unbound drug concentration into a mechanistic static model.^22^ Furthermore, the intracellular unbound concentration can be used as a scaling factor to explain differences in CYP enzyme inhibition in both microsomes and hepatocytes.^23^

In this study, we investigated the discordance in CL_int_ between microsomes and hepatocytes for certain drugs. We (1) considered the influence of inter-individual variability by studying the metabolic clearance of five commonly used probe drugs for CYP activity in 15 donor-matched human liver microsomes (HLM) and hepatocytes (HH). We also compared the protein composition of human liver (HL) homogenates, HLM, and HH from the same donors, to (2) elucidate whether the specific CYP amount in the two systems explained the differences in drug metabolism. Finally, (3) we investigated whether the concept of intracellular unbound drug concentration (as measured by Kp_uu_) could explain the differences in metabolic clearance.

## Methods

### HL Tissue

Excess tissue from HL resection surgery was obtained from the Department of Surgery, Uppsala University Hospital, Sweden. All 15 donors provided informed consent, in agreement with the approval from the Uppsala Regional Ethical Review Board (Ethical Approval no. 2009/028). The donors had a mean age of 65 years (ranging from 39 to 79 years) and a mean BMI of 26.4 kg/m^2^ (ranging from 20.1 to 32.9 kg/m^2^). Donor characteristics are summarized in Table S1.

Small pieces were immediately snap-frozen in methyl butane on dry ice and ethanol and stored at −150 °C. A larger piece was perfused with HypoThermosol FRS to remove the blood and kept on ice for at most 2 h prior to isolation of hepatocytes.

### Hepatocyte Isolation, Cryopreservation, and Thawing

Primary hepatocytes (HH) were isolated individually from each donor based on a two-step collagenase perfusion technique, as previously described.^24^ Isolated HH were resuspended and frozen at 10 × 10^6^ viable cells/mL in either KaLy–Cell medium (KaLy–Cell) or CryoStor CS10 (BioLife Solutions) with 10% FBS, as previously described.^25^ Cells were thawed at 37 °C for approximately 2 min, and dead cells were separated by centrifugation at 100*g* for 10 min at room temperature in Dulbecco’s modified Eagle medium (Gibco) with 30% isotonic Percoll (GE Healthcare), prior to use.

### Subcellular Fractionation and Microsomal Preparation

HLM were prepared from snap-frozen liver tissue pieces from each of the 15 donors, based on a previously described protocol.^26,27^ Briefly, liver pieces were thawed on ice and homogenized using a Potter-Elvehjem pestle at 2000 rpm in sucrose buffer (0.32 M sucrose, 10 mM Trisma base, 0.37 mg/mL EDTA, and complete mini protease inhibitor cocktail, pH 7.4). For each donor, an aliquot of the HL homogenate was collected, frozen, and saved for proteomics analysis. The remaining liver homogenate was centrifuged for 10 min at 7400*g*, 4 °C. The supernatant was transferred to a new tube, and the obtained pellet was collected, frozen, and saved for proteomics analysis. The supernatant was further centrifuged for 60 min at 104,000*g*, 4 °C, and the remaining pellet was resuspended in a buffer containing 0.25 M sucrose, 10 mM HEPES, and 0.8 mg/mL EDTA (pH 7.4). The resuspended microsomal fraction was frozen and kept at −80 °C. Protein yields after tissue homogenization and microsomal preparation are shown in Table 1.

**Table 1:** Protein Yield and Protein Amount Used in Incubations

	median	range
Yield Homogenization of Liver Tissue		
total protein per g liver (mg/g)	97.2	56.4–116.9
Yield Microsomal Preparation		
total microsomal protein per g liver (mg/g)	21.7	10.8–80.3
Total Protein in Incubations		
total microsomal protein (mg)	0.25	
hepatocytes, 10^6^ cells (mg)	0.84	0.23–1.68

### Protein Quantification

HL, HH, HLM, and the pellet from the first centrifugation (“discard pellet” obtained from 10 min centrifugation of the homogenate at 7400*g*, 4 °C) were lysed in 100 mM Tris–HCl buffer, pH 7.4, containing 2% SDS and 50 mM DTT. Proteins were denatured at 95 °C. Samples were prepared for proteomic analysis using the multi-enzyme digestion filter-aided sample preparation protocol, in which proteins are digested with LysC and trypsin.^28^ Protein and peptide amounts were determined based on tryptophan fluorescence.^29^ Peptides were separated on a reverse-phase EASY-spray LC column (2 μm C_18_ particles, 50 cm × 75 μm inner diameter; Thermo Fisher Scientific) using a 2 h acetonitrile gradient in 0.1% formic acid at a flow rate of 300 nL/min. The LC was coupled to a Q Exactive HF mass spectrometer (Thermo Fisher Scientific) operating in a data-dependent mode with survey scans at a resolution of 240,000, AGC target of 3 × 10^6^, and maximum injection time of 20 ms. The top 15 most abundant isotope patterns were selected from the survey scan with an isolation window of 1.4 *m*/*z* and fragmented with normalized collision energy (nCE) at 28.5. The MS/MS analysis was performed with a resolution of 15,000, AGC target of 1 × 10^5^, and maximum injection time of 60 ms. The resulting MS data were processed with MaxQuant,^30^ in which proteins are identified by searching MS and MS/MS data of peptides against the human UniProtKB. Carboamidomethylation was set as fixed modification and protein discovery rates were specified as 0.01. Spectral raw intensities were normalized with variance stabilization (vsn)^31^ and were subsequently used to calculate the protein concentrations using the Total Protein Approach.^32^

### Intrinsic Clearance (CL_int_) Measurements

Thawed HH from each of the 15 donors were resuspended to 1 million cells/mL (protein amount in Table 1) in Hepatocyte Maintenance Medium (Lonza) containing 10 μg/mL insulin, 5.5 μg/mL transferrin, 5 ng/mL selenium, 0.1 μM dexamethasone, 100 U/mL penicillin, and 100 μg/mL streptomycin. HLM, from each of the same 15 donors, were diluted to 0.5 mg/mL (Table 1) in 100 mM potassium phosphate buffer (80% K_2_HPO_4_ and 20% KH_2_PO_4_, pH 7.4). Metabolic activity reactions in HLM were initiated with 1 mM NADPH. The HH and HLM were each incubated with probe drugs to monitor specific CYP enzyme activities. The drugs were added as a cocktail containing 1 μM midazolam (CYP3A4/5), bufuralol (CYP2D6), bupropion (CYP2B6), and diclofenac (CYP2C9). In a separate incubation, CYP2C19 activity was monitored using 1 μM of omeprazole. The total incubation time was 90 min at 37 °C with shaking at 350 rpm. Aliquots were taken after 0, 5, 10, 15, 20, 30, 60, and 90 min, and the reactions were stopped by mixing with ice-cold acetonitrile/water (60:40); 50 nM warfarin was used as an internal standard. Compounds were quantified using UPLC–MS/MS, as described below. Clearance of the respective compound was determined using a substrate depletion method.^4^ Timepoint 0 represents 100% of the amount of the parent compound, and the remaining amount at each time point is converted to a percentage of this. The slope from the linear regression (*k*) of log percentage remaining and incubation time was used to calculate the intrinsic clearance (CL_int_)
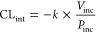
1or
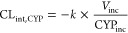
2where *V*_inc_ is the incubation volume, *P*_inc_ is the amount of total protein in the HLM [as determined using the BCA Protein Assay Reagent Kit (Thermo Fisher Scientific Inc.)] or million cells in HH incubation, and CYP_inc_ is the amount (pmol) of specific probe CYP protein in the HH or HLM incubation determined, as described under the “Protein Quantification” section. Clearance in HH and HLM (both determined with million cells or total amount of protein—CL_int_,_hep_ and CL_int,mic_—and with specific amount of probe CYP protein—CL_int,hep,CYP_ and CL_int,mic,CYP_) was corrected for unspecific binding (CL_int,u,hep_ and CL_int,u,mic_ or CL_int,u,hep,CYP_ and CL_int,u,mic,CYP_) by dividing CL_int_ with *f*_u,hep_ or *f*_u,mic_, respectively, as previously described.^33^

### Intracellular and Microsomal Compound Binding

Compound binding to the cell homogenate or microsomal fraction was determined using dialysis in the cassette mode, as previously described.^21^ Briefly, the cell homogenate or microsome fraction was spiked with the compounds and dialyzed for 4 h at 37 °C using a Rapid Equilibrium Dialysis device (Thermo Fisher Scientific Inc.). Protein was precipitated with acetonitrile/water (60:40) spiked with 50 nM warfarin, and samples were analyzed using UPLC–MS/MS, as described below. The fraction of unbound compound in the cell homogenate (*f*_u,hom_) or microsomal fraction (*f*_u,mic_) was calculated, as previously described^21^
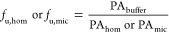
3where PA_buffer_ is the peak area of compound in the buffer chamber and PA_hom_ or PA_mic_ is the peak area of compound in the homogenate or microsomal chamber, respectively, all corrected for the peak area of the internal standard. The fraction of unbound compound in hepatocytes (*f*_u,cell_) was calculated according to
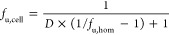
4where *D* was estimated for each homogenate preparation based on a cellular volume of 6.5 μL/mg protein,^34^ and on the protein concentration measured using the BCA protein assay reagent kit. The *f*_u,hep_ used for CL_int,u,hep_ was calculated as *f*_u,cell_ but with *D* being 0.1 corresponding to 10 times higher cell concentration in the binding experiment (10 × 10^6^ hepatocytes/mL) than that in the intrinsic clearance measurement.

### Intracellular Compound Accumulation

The compound accumulation in HH was determined after 15 and 30 min incubations. Since the accumulation was near-identical for both time points, their average value was used for all compounds, except diclofenac. For diclofenac, only the 15 min time point was used due to its rapid metabolism. An aliquot was collected at each time point, and cells were separated from the medium by 5 min centrifugation at 100*g* at 4 °C. Medium was collected, and cells were washed once with ice-cold PBS and collected after 5 min centrifugation at 100*g* at 4 °C. Compounds were released from medium and cells into acetonitrile/water (60:40) spiked with 50 nM warfarin and analyzed using UPLC–MS/MS, as described below. The ratio between the compound concentrations in the cells and medium (Kp) was calculated, as previously described^21^
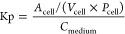
5where *A*_cell_ is the amount of compound in the cell samples, *V*_cell_ is the cellular volume (calculated based on the constant 6.5 μL/mg protein^34^ and the protein amount in the hepatocytes for each experiment), and *C*_medium_ is the compound concentration in the medium.

### Intracellular Unbound Drug Concentration

The intracellular unbound drug concentration in HH was calculated from the intracellular drug accumulation (Kp) and fraction unbound in the cell (*f*_u,cell_) by
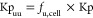
6

### Compound Quantification

Samples were centrifuged for 20 min at 2465*g* at 4 °C, and the compounds in the supernatant were analyzed by UPLC–MS/MS, consisting of a Waters Xevo TQ MS with electrospray ionization coupled to a Waters Acquity UPLC. Compounds were separated with a 1.8 min gradient elution of acetonitrile and 0.1% formic acid (flow rate 0.5 mL/min) on a Waters BEH C18 column, 2.1 × 50 mm (1.7 μm) at 60 °C (see Table S2 for LC–MS/MS conditions).

### Scaling CL_int,u_ to *In Vivo* CL_int,mic_ and CL_int,hep_ (mL/min/kg Body Weight)

To investigate factors that could influence the CL_int_ calculated for HH and HLM, three different ways of scaling the *in vitro* clearance to mL/min/kg body weight (mL/min/kg bw) were tested.

(1) CL_int,u_ values from HH and HLM (eq 1, mL/min/mg protein) were scaled to mL/min/kg bw with literature scaling factors:

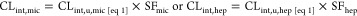
7where (2,4,5,7,35) and .^1,2,5,7^

(2) CL_int,u_ values from HH and HLM normalized against the specific probed CYP (eq 2, mL/min/pmol CYP) were scaled to mL/min/kg bw using the amount of the corresponding CYP in the liver:

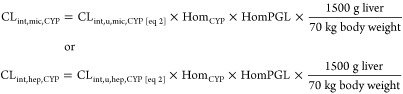
8where CL_int,u,mic,CYP_ and CL_int,u,hep,CYP_ are the clearance measured in HLM and HH, respectively, per amount of specific probe CYP protein (mL/min/pmol CYP). Hom_CYP_ is the specific probe CYP concentration in HL (pmol CYP/mg total protein) determined, as described under the “Protein Quantification” section, and HomPGL is the mg total protein in the liver homogenate per gram liver tissue (92 mg total protein/g liver determined from the 15 donors from the “Subcellular Fractionation and Microsomal Preparation” section).

(3) The CL_int,mic,CYP_ was adjusted for the intracellular unbound concentrations (Kp_uu_) from HH by:

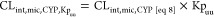
9

### Statistical Analysis

Average fold difference (AFD) and absolute AFD (AAFD) were calculated by
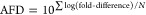
10
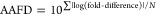
11where the fold difference is the ratio between each comparison and *N* denotes the number of comparisons carried out in the analysis.^36^

Proteins that were identified with at least three peptides were considered for the bioinformatics analysis. Principal component analysis (PCA) was performed using SIMCA (Sartorius Stedim Biotech), version 15.0.0.4783. Enriched proteins were determined using *t*-test with a permutation-based FDR calculation (implemented in Perseus,^37^ version 1.6.2.3). Functional annotation clustering of GOBP, GOCC, and KEGG terms was performed with David, version 6.8, using default settings.^38^ Enriched protein functions were analyzed using Proteomaps.^39^ Proteins were annotated with subcellular locations from the “Subcellular location” data from the Human Protein Atlas (HPA),^40^ where proteins were classified in the following groups “ER,” “plasma membrane,” “mitochondria,” “Golgi apparatus,” “cytosol,” and “nucleus” (including nucleoplasm, nuclear speckles, nuclear membrane, nuclear bodies, nucleoli, nucleoli fibrillary center, and nucleus). Proteins localized in several subcellular groups in the HPA were annotated to all of these subcellular compartments. Statistical analysis and figures were made using GraphPad Prism, version 7.03, and Excel. Pearson’s correlation coefficients were calculated from logarithmic values.

## Results

### Comparison of Metabolic Activity in HLM and HH from 15 Matched Donors

**Figure 1:**
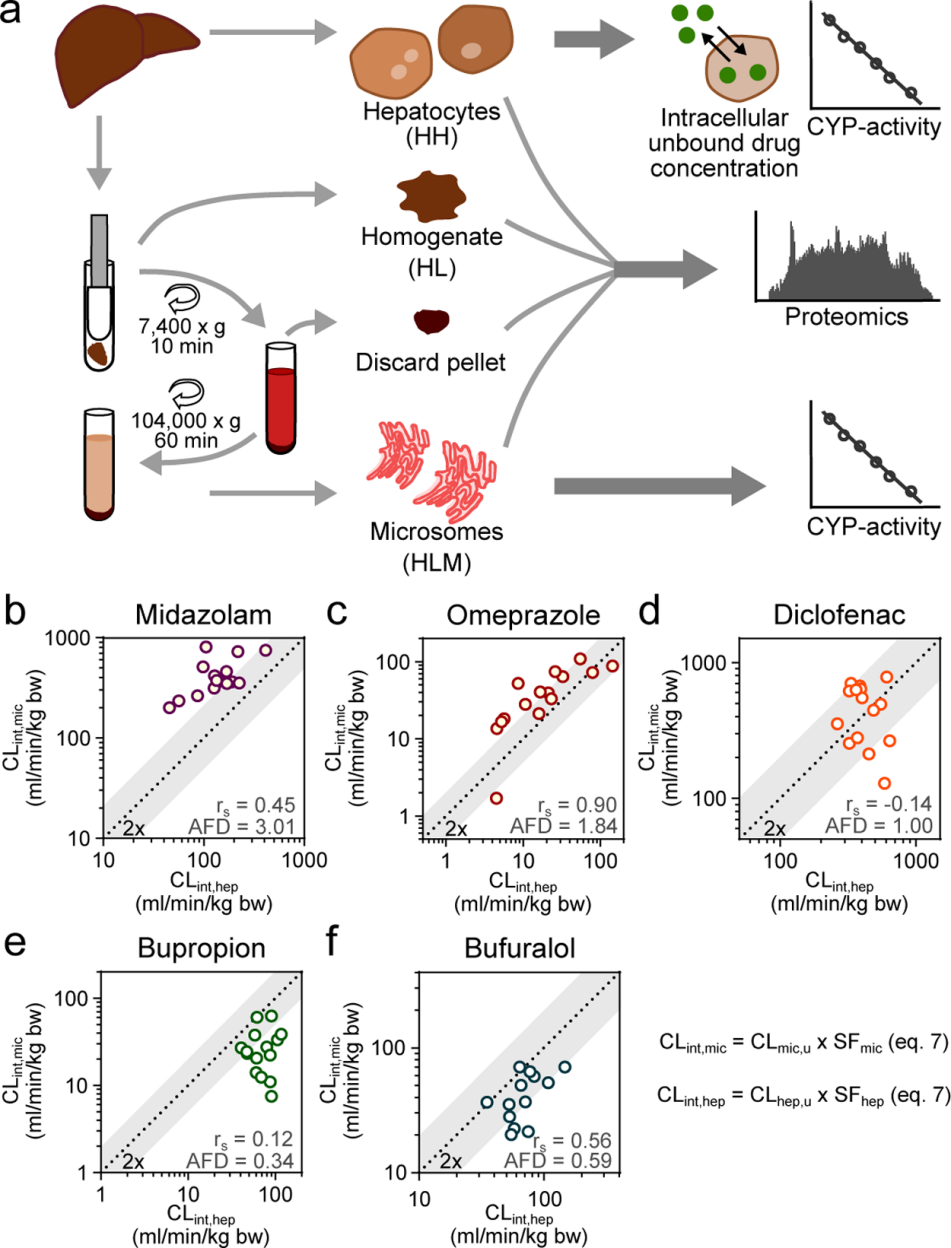
Metabolic activity of drug metabolizing enzymes in human liver microsomes (HLM) and hepatocytes (HH). (a) Workflow of the collection of the different sample types (HH, HL, discard pellets, and HLM), and which experiments were conducted using the respective sample type. (b–f) Intrinsic clearance of five probe CYP substrates (midazolam, omeprazole, diclofenac, bupropion, and bufuralol) measured in 15 donor-matched HLM and HH. Unbound *in vitro* clearance was scaled to kg body weight with eq 7. *r*_s_ = Spearman’s rank correlation coefficient and AFD = average fold difference (eq 10).^36^

Most *in vitro* drug metabolism studies use HLM and HH derived from different and pooled donor batches.^1,2,7^ While this gives a good estimate of the drug clearance for the average population, it does not reflect inter-individual differences. Also, direct comparisons of the systems are not possible. To address this limitation, the first step of our analysis was to investigate the influence of inter-individual variability on the differences in intrinsic clearance (CL_int_) between the two systems. For this purpose, we produced HLM and HH from liver samples obtained from the same 15 human donors (Figure 1a) and analyzed the clearance of five probe CYP substrates (bupropion—CYP2B6, diclofenac—CYP2C9, omeprazole—CYP2C19, bufuralol—CYP2D6, and midazolam—CYP3A4; Figure 1b–f).

We noted a generally higher CL_int_ in HLM for midazolam (AFD = 3.01) and omeprazole (AFD = 1.84) across the 15 donors (calculated from eq 7). The median midazolam CL_int,mic_ was 375 mL/min/kg bodyweight (bw) (range 203–819 mL/min/kg bw) compared to the median CL_int,hep_ of 134 mL/min/kg bw (45–414 mL/min/kg bw). The median omeprazole CL_int,mic_ was 40 mL/min/kg bw (2–109 mL/min/kg bw) compared to the median CL_int,hep_ of 17 mL/min/kg bw (5–144 mL/min/kg bw; Figure 1b,c, Table S3; and Figures S6 and S7).

In contrast, the CL_int_ values of bupropion and bufuralol were generally higher in HH across the 15 donors (AFD = 0.34 and 0.63, respectively), where the median bupropion CL_int,mic_ was 24 mL/min/kg bw (8–62 mL/min/kg bw) compared to the median CL_int,hep_ of 69 mL/min/kg bw (41–118 mL/min/kg bw). The median bufuralol CL_int,mic_ was 37 mL/min/kg bw (20–70 mL/min/kg bw) compared to median bufuralol CL_int,hep_ of 66 mL/min/kg bw (22–146 mL/min/kg bw; Figure 1e,f, Table S3; and Figures S8and S10).

The CL_int_ of diclofenac was in general similar in HLM and HH (AFD = 0.99), with median diclofenac CL_int,mic_ of 494 mL/min/kg bw (129–782 mL/min/kg bw) compared to the median CL_int,hep_ of 392 mL/min/kg bw (263–637 mL/min/kg bw; Figure 1d, Table S3; and Figure S9).

With these systematic differences in the CL_int_ of HLM and HH from matching donors, we ruled out inter-individual variability in drug metabolism of these probe substrates as the reason. We propose that the differences are rather an artifact of improper scaling.

### Comparison of the Protein Profiles of HLM, HH, and HL

**Figure 2:**
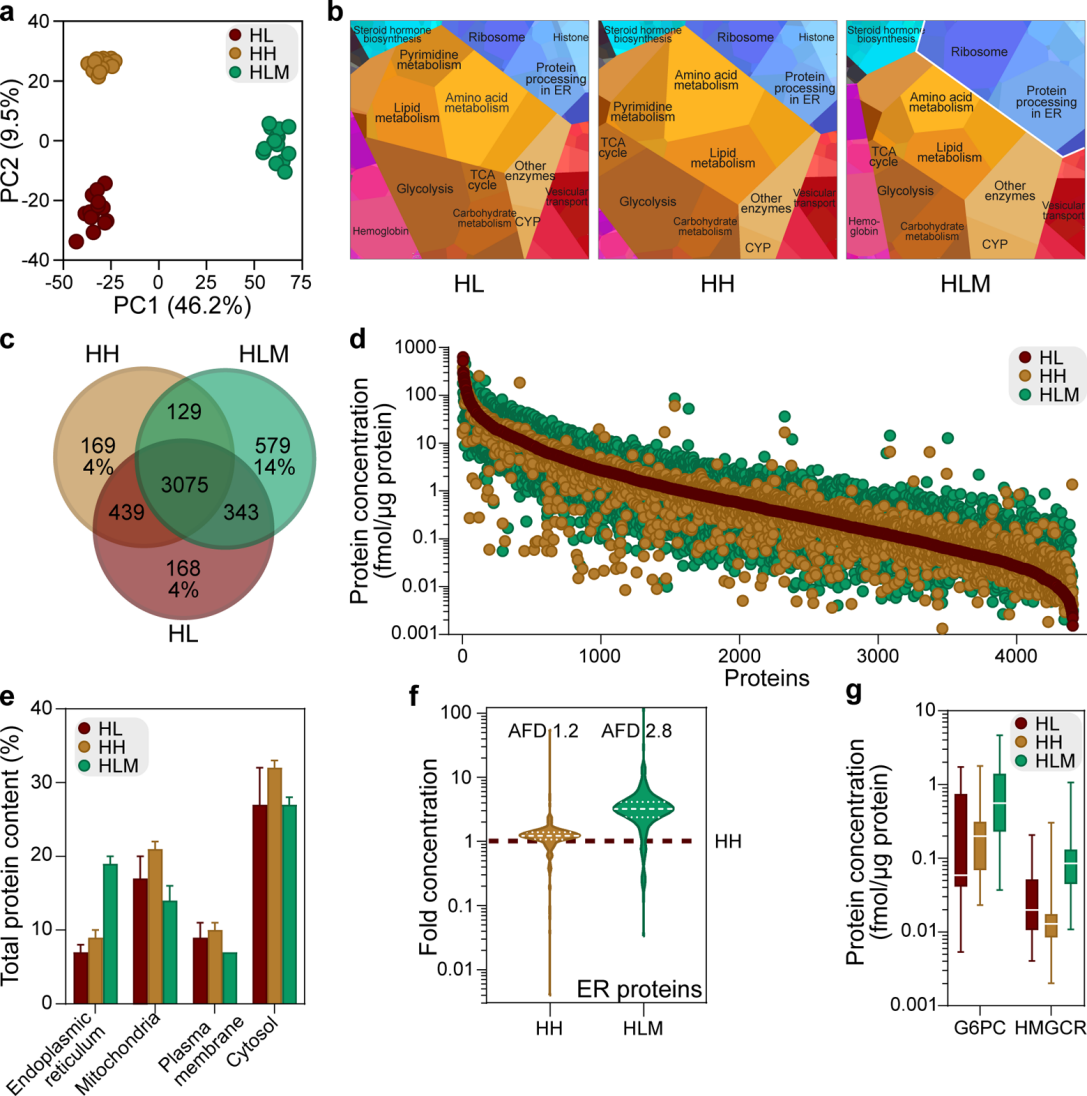
Global proteomics analysis of human liver (HL) homogenates, isolated hepatocytes (HH), and liver microsomes (HLM). (a) PCA of proteins in HL, HH, and HLM from 15 donors. (b) Proteomaps^39^ displaying the quantitative composition of protein function in the proteomes of the three sample types, using average concentrations from the 15 donors. The size of each polygon indicates the abundance of proteins involved in the cellular function according to the KEGG pathway. (c) Overlap of quantified proteins in each sample type. (d) Range of concentrations of overlapping proteins in each sample type, based on average concentrations from the 15 donors. Proteins are ranked based on the median concentration in the HL. (e) Proportion of the total protein content in different subcellular locations (proteins annotated by HPA^40^). Bars show average levels, and error bars denote standard deviation for the 15 donors. (f) Distribution of fold concentrations of ER-located proteins in HH and HLM compared to HL. Dashed and dotted white lines denote median, and upper and lower quartiles, respectively. (g) Protein concentrations of historically used activity markers in HLM for ER in the three sample types. The lines shows median values and whiskers minimum and maximum values from the 15 donors. AFD, average fold difference.^36^

In scaling *in vitro* clearance from HLM to mL/min/kg bw, a standard yield of mg microsomal protein per gram liver (MPPGL) is commonly used as a scaling factor (as used in eq 7).^2,4,7^ Although this commonly applied MPPGL is corrected for microsomal recovery of certain proteins (e.g., by accounting for activity measurements of microsomal markers^41,42^), the microsomal fraction contains all proteins obtained in the fraction collected during the subcellular processing. Therefore, microsomal recovery may not reflect differences in amounts of the actual proteins involved in the metabolic activity. To better understand the differences in protein composition in HLM, HH, and homogenate of HL, we used global proteomics analysis (Figure 1a). The complete data sets from the matched donors are available in the Supporting Information (Data S1). Both PCA and functional analysis of the proteomes using Proteomaps^39^ showed that the overall protein composition of HLM differed from both the HH and HL (Figure 2a; Figure 2b). Biosynthesis processes (amino acid metabolism, glycolysis, carbohydrate metabolism, and lipid metabolism) dominated in all three sample types, but the HLM Proteomap had the largest proportion of proteins related to the ER, where CYP enzymes are located (Figure 2b). Of 3989 proteins, 3075 (77%) were the same for the three systems (Figure 2c). Despite this large overlap in the *identity* of the quantified proteins, the protein *expression levels* of HLM differed from both the HL (AAFDs of 3.0) and HH (AAFD 3.1; Figure 2d). HLM proteins had significantly higher concentrations (Figure S1a; FDR = 0.01 and S0 = 2) of proteins involved in ER-associated pathways, such as fatty acid and drug metabolism (Data S2). This is in line with that HLMs are considered to be vesicles derived from the ER.^12^

Since we found such a big overlap in proteins in HLM, HH, and HL, we investigated the fractional contribution (% total protein content) of proteins from different subcellular locations.^40^ This analysis confirmed that ER-annotated proteins made up a larger proportion of the total protein content in the HLM (19%) than that in the HL (7%) and HH (9%; Figure 2e). However, the HLM also contained proteins associated with other subcellular compartments, including mitochondria (14% of the total HLM protein content, compared to 17 and 21% in HL and HH, respectively). The proportion of cytosolic proteins was comparable for HLM (27%), HL (27%), and HH (32%). However, HLM also contained a large proportion of nuclear proteins (19%) that are expected to be captured by the first low-speed centrifugation pellet^43−46^ (the “discard pellet”; Figure 1a). In fact, the fraction of nuclear proteins in HLM was comparable to that in the discard pellets (which contained 20% nuclear proteins) and not much lower than either HL (28%) or HH (24%; Figure S1b–d). This demonstrates that the HLM fractions are “contaminated” with many proteins that are not associated with the ER compartment, an observation supported by previous investigations.^18,19^

We further investigated the enrichment of ER-annotated proteins. As with the complete set of quantified proteins, the HLM, HL, HH, and discard pellet all contained substantial concentrations of ER-associated proteins (Figure S1e–h). The ER-related proteins were enriched in general 2.8-fold (Figure 2f,g) in the HLM but with a large variability ranging from 0.03 to 120-fold for the different proteins.

### Enrichment of CYP Enzymes in HLM

**Figure 3:**
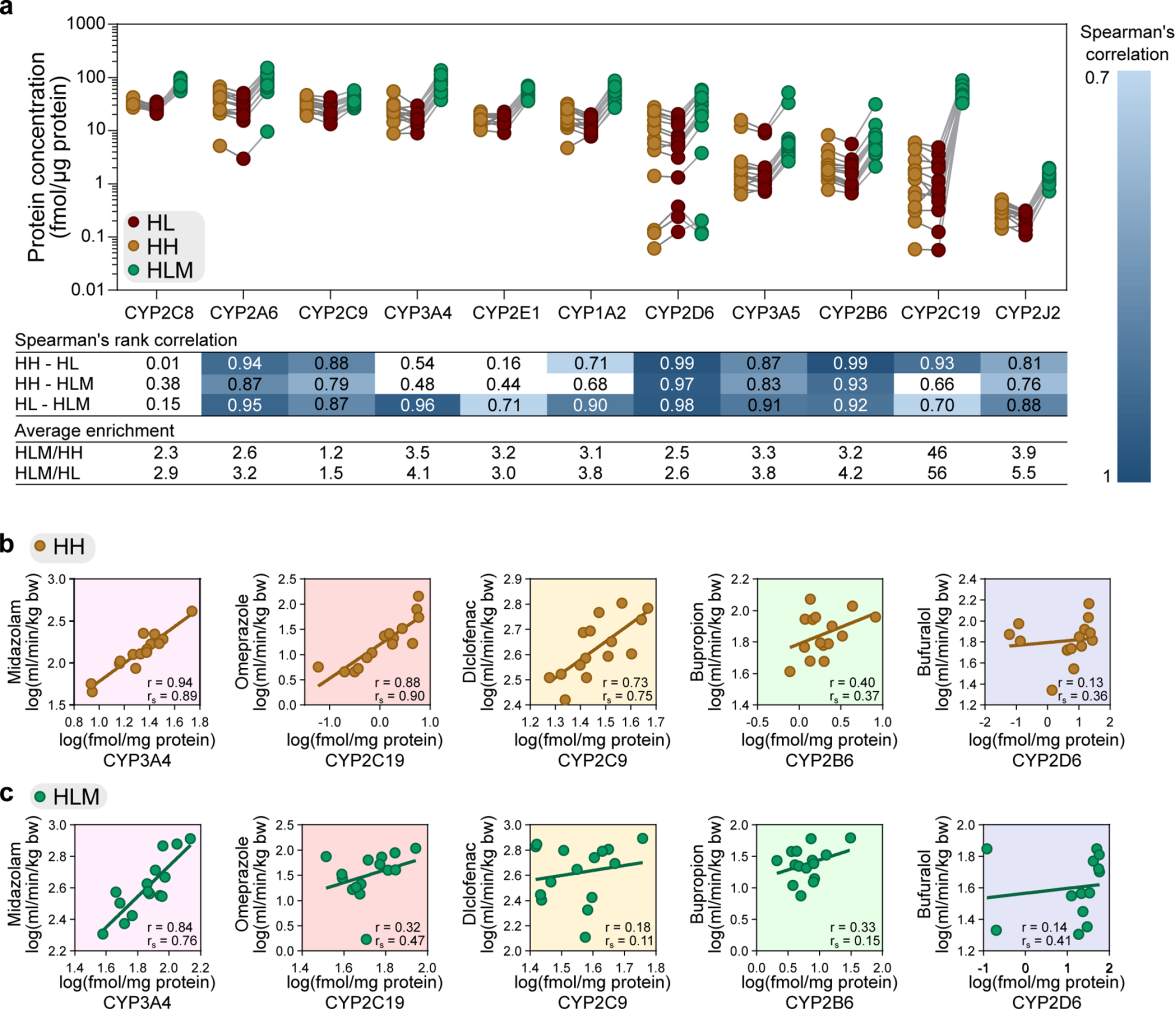
Protein concentration and metabolic activity of drug metabolizing enzymes. (a) Protein concentrations of CYP enzymes in liver homogenate (HL), isolated hepatocytes (HH), and liver microsomes (HLM) from the 15 donors. Concentration levels are given in fmol/μg total protein. Spearman’s rank correlations (*r*_s_) compare the relative expression of each enzyme across the donors between the sample type, with significant correlation coefficients >0.7 (*p* < 0.006, after Bonferroni correction for multiple comparisons). Average enrichment of HLM compared to HL and HH was calculated based on concentrations from the 15 donors. (b,c) Comparison of metabolic activity (CL_int,hep_ and CL_int,mic_, eq 7) of the probe substrates and protein concentrations of the corresponding probe CYP enzymes in the donor-matched HH and HLM. *r* = Pearson’s correlation coefficient calculated from the log-transformed values and *r*_s_ = Spearman’s rank correlation coefficient.

The variability in enrichment for the supposedly ER-localized proteins in HLM made us suspect that the HLM preparation procedure itself might be responsible for the variability in CYP enzyme levels. This in turn would be reflected by variability in measurements of metabolic activity. The median concentrations of the most important drug metabolizing CYP enzymes^47^ in HLM ranged from 1.3 (CYP2J2) to 76.5 (CYP2C8) fmol/μg protein (Figure 3a) for the 15 donors. Similar to the other ER-annotated proteins, the average enrichment of CYP enzymes was 3.2-fold higher in the HLM than that in the HL and HH (HL and HH CYP levels were in good agreement with previous reports; Figures 3a and S2b,c(48−50)). However, the CYP enzymes were enriched to different degrees in HLM than that in HL and HH, ranging from 1.2 (CYP2C9) to 56 (CYP2C19)-fold. The rank order of donors was in general similar to the three sample types [median Spearman’s rank correlation (*r*_s_) of 0.87; Figure 3a and Table S3]. This indicates that the procedure for preparing HLM and HH samples preserved the order in the liver samples (i.e., donors with the highest specific CYP concentrations in HL also showed the highest concentrations in HLM and HH). For CYP3A4 and CYP2C19 (probed by midazolam and omeprazole, respectively), the rank correlations between protein concentrations in HLM and HH were weaker (*r*_s_ = 0.48 and *r*_s_ = 0.66, respectively; Figure 3a and Table S3), which could potentially translate to differences in metabolic clearance.

### Impact on Concurrence Between HLM and HH CL_int_ from Normalization with the Amount of Specific CYP

To investigate whether variable enrichment of CYP enzymes contributed to the differences in CL_int_ from HLM and HH, we normalized CL_int_ with the amounts of individual CYPs probed by each of the five substrates, instead of using total protein (HLM) or number of cells (HH) (eq 8; giving mL/min/pmol CYP). This normalization for CYP3A4 resulted in more similar CL_int,CYP_ values for midazolam in HLM and HH, with the AFD improving from 3.01 to 1.62 (Table 2; Table S3; and Figure S11).

**Table 2:** AFD and Correlation Parameters from Comparisons of CL_int,mic_ and CL_int,hep_ Calculated with Different Scaling Factors^a^

		literature scaling factors (eq 7)	proteomics scaling factors (eq 8)	proteomics and Kp_uu_ scaling factors (eq 9)
midazolam	AFD	3.01	1.62	0.52
	*r*_s_	0.45	0.66	0.66
	*r*	0.64	0.78	0.82
omeprazole	AFD	1.84	0.08	0.12
	*r*_s_	0.90	0.92	0.90
	*r*	0.77	0.87	0.87
diclofenac	AFD	1.00	1.55	1.11
	*r*_s_	–0.14	0.28	0.41
	*r*	–0.22	0.21	0.39
bupropion	AFD	0.34	0.20	0.24
	*r*_s_	0.12	0.08	–0.03
	*r*	0.00	0.01	–0.01
bufuralol	AFD	0.59	0.42	1.08
	*r*_s_	0.56	0.68	0.62
	*r*	0.52	0.73	0.67

a *r* = Pearson’s correlation coefficient calculated from log-transformed values, *r*_s_ = Spearman’s rank correlation coefficient, and AFD = average fold difference.

However, for the other four compounds, normalization with the respective CYP probe (eq 8) resulted in larger differences in CL_int_ than normalization to mg protein or million hepatocytes (eq 7; Table 2; Table S3; and Figure S11). For diclofenac, AFD increased from 1.00 to 1.55. Meanwhile, AFDs were reduced for bupropion (from 0.34 to 0.2), omeprazole (from 1.84 to 0.08), and bufuralol (from 0.59 to 0.42). Notably, three outliers in the omeprazole correlation had surprisingly comparable unadjusted CL_int_ in HLM and HH, despite 42-fold to 228-fold higher CYP2C19 levels in HLM. In contrast, bufuralol CL_int_ could not be determined in the HLM preparations of two donors due to undetectable depletion; meanwhile, relatively high CL_int_ (26 and 170 mL/min/kg bw; eq 8) was obtained in the corresponding HH. These findings suggest that metabolic pathways other than the probed CYP enzymes contribute to the CL_int_ of omeprazole and bufuralol in the HH.

In contrast to the mostly increased overall CL_int_ differences between HLM and HH in absolute values (AFD), the rank order agreement for the 15 donors improved for three of the compounds by compensating for specific CYP content: midazolam (*r*_s_ from 0.45 to 0.66), diclofenac (*r*_s_ from −0.14 to 0.28), and bufuralol (*r*_s_ from 0.56 to 0.68). The rank order was unaffected though for omeprazole (*r*_s_ from 0.90 to 0.92) and bupropion (*r*_s_ from 0.12 to 0.08). Accordingly, the explained variance (Pearson *r*^2^) improved by taking CYP content into account. Thus, compensating for CYP amounts tended to cancel out some of the variability of the two experimental systems, while simultaneously introducing an offset in the CL_int_ values.

But why did the compensation for CYP amount not consistently improve the correspondence in absolute values (AFD) for HLM- and HH-derived CL_int_? We further examined the correlations between CL_int_ and the CYP concentrations for each of the five probe substrates. For midazolam, CL_int_ (CL_int,mic_ and CL_int,hep_ per mg protein and million hepatocytes; eq 7) correlated well with the CYP3A4 concentration in both HLM [Pearson’s correlation coefficient (*r*) = 0.84) and HH (*r* = 0.94; Figure 3b,c]. A high correlation was also found in HH between both omeprazole CL_int,hep_ and CYP2C19_HH_ concentrations (*r* = 0.88) and diclofenac CL_int,hep_ and CYP2C9_HH_ concentrations (*r* = 0.73). Correlations were lower in HLM for both probe substrates (omeprazole CL_int,mic_—CYP2C19_HLM_, *r* = 0.32; diclofenac CL_int,mic_—CYP2C9_HLM_, *r* = 0.18; Figure 3b,c). (Notably, the correlations for omeprazole CL_int,mic_—CYP2C19_HLM_ were strongly influenced by one outlier, which was also an outlier in the HLM and HH correlation of CYP adjusted CL_int_. Without this outlier, the correlation improved to *r* = 0.49). Interestingly, these two enzymes—both belonging to the subfamily CYP2C—were very differently enriched in the HLM. CYP2C9, with higher HL concentrations, was poorly enriched (AFD 1.4-fold) in the HLM, while CYP2C19, with lower HL concentrations, was 50-fold more concentrated in the HLM than that in either HL (56-fold) or HH (46-fold; Figure 3a; Table S3). In line with this, global analysis of the proteomics data showed that the proteins with higher initial HL concentrations were less enriched in the HLM (*r*_s_ = −0.33; Figure S3e).

For bupropion, lower correlations between CL_int_ and CYP2B6 concentration were obtained in both HLM and HH (CL_int,mic_–CYP2B6_HLM_, *r* = 0.33; CL_int,hep_–CYP2B6_HH_, *r* = 0.40; Figure 3b,c). Low correlations were also observed between bufuralol CL_int_ and CYP2D6 concentrations in both systems (CL_int,mic_–CYP2D6_HLM_, *r* = 0.14; CL_int,hep_–CYP2D6_HH_, *r* = 0.13; Figure 3b,c; Table 2; Table S3; and Figure S11). Notably, two donors had high bufuralol CL_int_ in both HLM and HH despite low CYP2D6 amounts, which further indicates that bufuralol is metabolized by enzymes other than CYP2D6 in the two systems.

Interestingly, the CL_int_ of the compounds also correlated well with levels of CYPs other than the ones they were probing. For instance, midazolam CL_int_ correlated well with the probed CYP3A enzymes, that is, CYP3A4 and CYP3A5, but it also correlated well with CYP1A2 levels in both HLM and HH. Similarly, omeprazole CL_int_ correlated well with the probed CYP2C19 in both HLM and HH. In addition, it also correlated well with CYP3A4, which metabolizes omeprazole to some extent,^51^ and with CYP1A2 levels (Figure S4a,b; Table S4) in both systems. This implies that several enzymes may be involved in the metabolism of these compounds and affect CL_int_ in both experimental systems.

### Impact on Concurrence between HLM and HH CL_int_ from Normalization by CYP Amount and Intracellular Unbound Drug Concentration (Kp_uu_)

**Figure 4:**
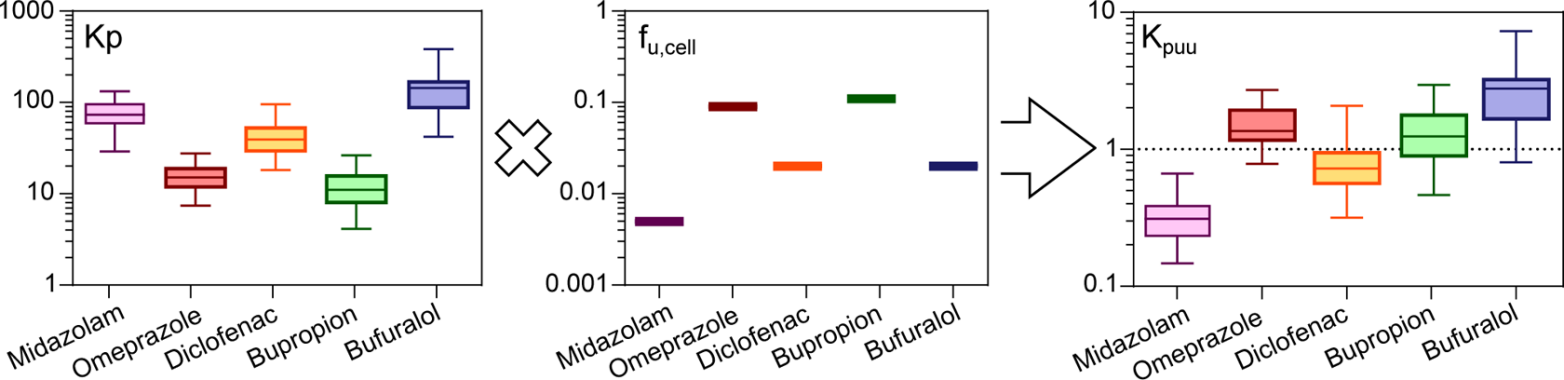
Intracellular unbound concentration of drugs in HHs. Accumulation (Kp), unbound fraction (*f*_u,cell_), and intracellular unbound drug accumulation (Kp_uu_) of probe CYP substrates in HH from 15 donors.

The amounts of probe-specific CYPs improved correlations but introduced a general shift in the CL_int_ values (i.e., increased AFD). We therefore investigated whether accounting for intracellular unbound drug concentrations (as measured by Kp_uu_) would correct these shifts. For this purpose, we determined Kp_uu_ for each of the five compounds in HH from the 15 donors. For omeprazole, bupropion, and diclofenac, Kp_uu_ varied close to 1 (median Kp_uu_ = 1.4, 1.2, and 0.7, respectively; Table 3; Figure 4; Table S3; and Figure S5b–d), indicating on average equal drug concentrations within and outside of the cell. Kp_uu_ was lower for midazolam (median Kp_uu_ = 0.3, range 0.2–0.7, Table 3; Figure 4; Table S3; and Figure S5a), indicating that unbound drug concentrations were lower inside than outside of the cells. In contrast, bufuralol concentrations were slightly elevated intracellularly (median Kp_uu_ = 2.8, range 0.8–7.3, Table 3; Figure 4; Table S3; and Figure S5e), indicating an accumulation of unbound drug in the cell.

**Table 3:** Intracellular Unbound Concentration of Drugs in Human Hepatocytes

	Kp_uu_		*f*_u,cell_
	median	range (min to max)^a^	median
midazolam	0.31	0.14–0.66	0.51
omeprazole	1.43	0.7–2.61	0.93
diclofenac^b^	0.73	0.31–2.07	0.83
bupropion	1.24	0.46–2.95	0.96
bufuralol	2.76	0.8–7.28	0.76

a Range across hepatocytes from 15 donors.

b Determined after 15 min incubation only.

**Figure 5:**
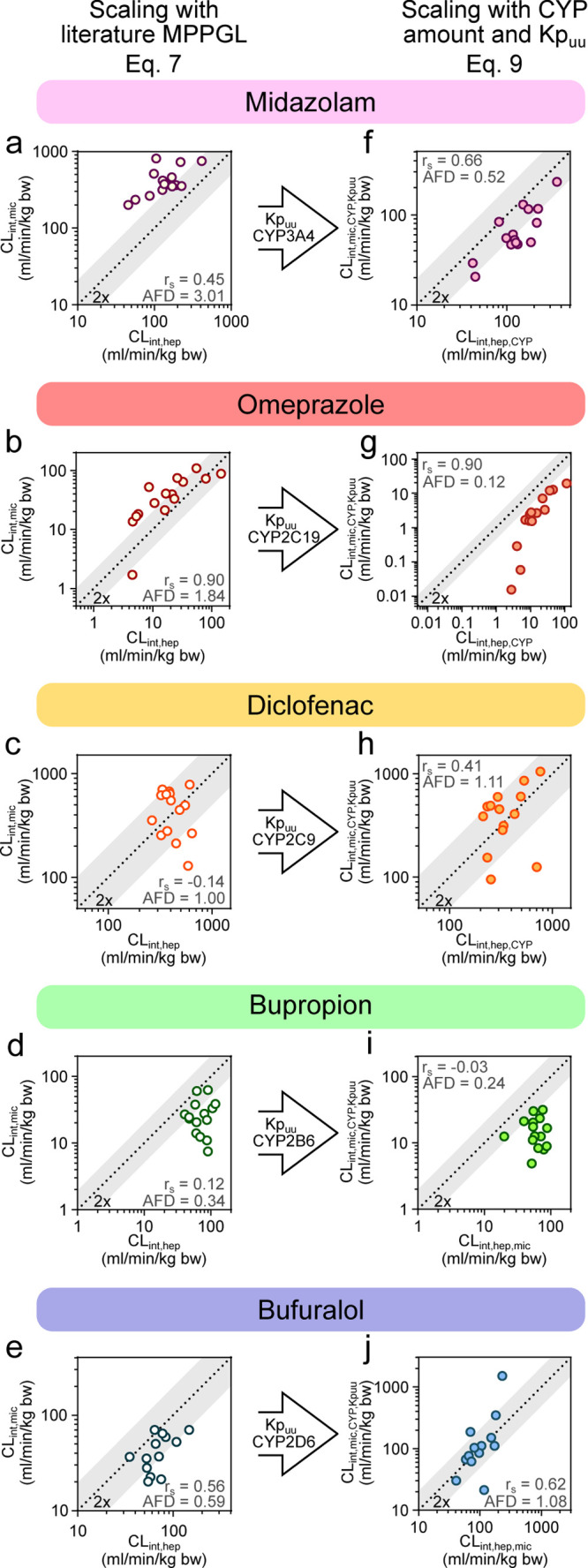
Adjustment of CL_int,mic_ and CL_int,hep_ with factors influencing metabolic clearance. (a–e) Intrinsic clearance of five probe CYP substrates (midazolam, omeprazole, diclofenac, bupropion, and bufuralol) measured in 15 donor-matched HLM and HH. Unbound *in vitro* clearance was scaled to kg body weight with eq 7. (f–j) Intrinsic clearance of the five probe CYP substrates after adjustment of Kp_uu_ and probe CYP amount in the respective system with eq 9. *r*_s_ = Spearman’s rank correlation coefficient and AFD = average fold difference.^36^

The Kp_uu_ from each of the 15 donors was then used to adjust the respective CYP normalized CL_int_ in HLM (CL_int,mic,CYP,Kpuu_; eq 9). This resulted in overall improvements in the correspondence between HLM- and HH-derived CL_int_ for four of the five compounds, although in some cases, the changes were minor (Figure 5a–j; Table 2; and Table S3).

Kp_uu_-adjustment improved the AFD from 0.42 to 1.08 for bufuralol (Kp_uu_ > 1; Figure 5e,j; Table 2; and Table S3) and from 1.55 to 1.11 for diclofenac (Kp_uu_ < 1; Figure 5c,h; Table 2; and Table S3). In contrast, the higher CYP-adjusted midazolam in HLM was over-compensated by the low intracellular unbound concentrations (median Kp_uu_ of 0.3), which reduced AFD from 1.62 to 0.52 (Figure 5a,f and Table 2). Since midazolam is one of the more rapidly metabolized compounds, Kp_uu_ may not accurately capture the constantly decreasing intracellular concentrations.

For bupropion and omeprazole, the near-unity Kp_uu_ suggested that predictions would not be greatly affected. Both compounds retained similar AFDs as before and after Kp_uu_ compensation (AFD improved marginally from 0.20 to 0.24 for bupropion and from 0.08 to 0.12 for omeprazole; Figure 5b,d,g,i; Table 2; and Table S3).

## Discussion

In this study, we sought to understand differences in metabolic activity obtained from the two major assay systems for drug metabolism studies, HLM and HH. To this end, we extensively investigated HLM and HH prepared from 15 matched donors. Specifically, we determined the metabolic CL_int_ of probe substrates of different CYP isoforms: CYP2B6 (bupropion), CYP2D6 (bufuralol), CYP2C9 (diclofenac), CYP2C19 (omeprazole), CYP3A4 (midazolam), and investigated factors that could influence the CL_int_, such as (1) inter-individual differences, (2) CYP amount, and (3) intracellular unbound drug concentrations (Kp_uu_). Furthermore, we quantified the global proteomes of HLM, HH, and the corresponding homogenated HL from the 15 matched donors. To the best of our knowledge, this is the first such donor-matched study.

### Influence of Inter-individual Variability on Drug Metabolic CL_int_

We could rule out that the disconnect typically observed between CL_int_ in HLM and HH in the literature^1,2,7,13^ is solely a result of inter-individual differences as our donor-matched samples still resulted in systematic differences in CL_int_ (AFD ranging from 0.34 to 2.95; Figure 1a–e, Table 2). This is in line with previous observations from a smaller-scale study.^52^ The fact that CL_int,mic_ was higher than CL_int,hep_ for midazolam (CYP3A4) and omeprazole (CYP2C19) was in agreement with previous observations.^2,7,13,53,54^ For diclofenac—where we obtained similar CL_int_ in HLM and HH—contradicting results have been reported previously, with CL_int_ either higher in HLM^7,54^ or in HH.^2,5^ The higher CL_int,hep_ of bufuralol was in line with that shown previously.^7^

The accuracy and appropriateness of the frequently used scaling factors for microsomes and hepatocytes (45 mg MPPGL and 120 × 10^6^ cells/g liver, respectively),^2,4,7,35,55^ that we used in our initial comparison of CL_int_ from the two systems (eq 7), have been extensively discussed.^3,56−58^ It has been suggested that individual scaling factors determined for each batch may improve CL_int_ predictions in HLM and HH.^3,41,56−58^ However, such scaling factors would still only consider the total activity in the two systems and not necessarily reflect differences in metabolic activity of specific enzymes. For instance, AFD in our study ranged from just below to just above unity for all five compounds [mean AFD between 0.34 (bupropion) and 2.96 (midazolam)]. Thus, selecting any common scaling factor would improve the predictions for some compounds while making others worse since changes to the scaling factor would move all data points in the same direction. This is in agreement with a previous observation where one scaling factor—calculated from the regression offset approach—was not sufficient for accurate *in vitro*–*in vivo* extrapolation of compounds with different metabolic profiles.^13^

Furthermore, although adjustments of the MPPGL scaling factor have been introduced to account for variable recovery of metabolic activity in the HLM preparations—for example using glucose-6-phosphatase activity or amount of total CYP enzymes as proxies^41,42^—this is still only a general measure of the HLM activity. Thus, its validity is based on the assumption that all microsomal proteins are recovered to the same extent as the activity markers, and any differences in the recovery of specific proteins are not taken into account.

### Global Proteomics Analysis of HLM, HH, and HL

Our global proteomics analysis confirmed that the HH samples had protein expression patterns similar to the ones of the HL samples from which they were isolated. This is as expected because HH is the dominating cell population in the liver, comprising approximately 80% of the liver volume.^59^ Furthermore, it indicates that the hepatocyte isolation procedure does not have a major effect on the HH proteome. In contrast, the HLM proteomes differed significantly from those of the corresponding HL and HH samples. HLM are considered as ER-derived vesicles^12^ and would therefore be expected to be enriched with ER-related proteins. However, the enrichment was lower than expected in HLM (on average 2.8-fold). As the ER-associated proteins constituted 7% of the total protein content in our HL—assuming complete isolation and full recovery of the ER fraction in the HLM—we expected a 14-fold enrichment. The less-than-complete enrichment of ER-associated proteins was also reflected in a 2.2 to 4.6-fold enrichment of the ER-membrane markers, CANX and POR, that is, similar or slightly lower than previous estimates (Figure S1i).^16^ The traditional ER-activity markers, glucose-6-phosphatase (G6PC) and HMG-CoA reductase (HMGCR),^60^ were also similarly enriched (3.4 to 7.4-fold) in the HLM (Figure 2g). Besides the ER-related proteins, we also found specific membrane markers for other organelles, for example, lysosomes, peroxisomes, and plasma membranes, in the HLM. These organelle marker proteins were enriched to a similar degree in our HLM, as shown previously with a targeted proteomics approach (Figure S1i).^16^ The pronounced variability in enrichment degree for different ER-related proteins (0.03 to 120-fold) demonstrates the complexity of the enrichment process and limits the use of specific protein markers as scaling factors.

The lower-than-expected enrichment could have multiple reasons. First, proteins from other subcellular compartments in the HLM could have diluted the ER-related proteins. In agreement with previous observations,^18^ the HLM contained proportions of proteins from the mitochondria, nucleus, and cytosol comparable to what is in the whole-cell HH and HL samples. Presumably, nuclei, cell debris, and mitochondria originating from homogenized cells would be collected in the resulting “discard pellet,” and thus lower proportions of these proteins would be found in the subsequent microsomal fractions obtained after centrifugation at 104,000*g* for 60 min.^43−46^ Such separation of unwanted cell material was confirmed since the nuclear proteins constituted a smaller proportion of the total protein content of the HLM than that in both HH and HL (on average 19% compared to 28 and 24%). Nonetheless, relatively large proportions of nuclear proteins contaminated the HLM and these nuclear proteins were not enriched in the discard pellet as commonly assumed. Rather, they were found in lower proportions than that in both HL and HH (20% as compared to 28 and 24%, respectively; Figure S1b). Furthermore, as mitochondria are divided into a heavy and a light fraction that sediment at 3000*g* and 15,000 to 17,000*g*, respectively,^45^ it is non-trivial to achieve a complete separation of this organelle by centrifugation.

Second, ER-related proteins can be lost in the first low-speed centrifugation step,^15,17^ thereby reducing their enrichment in the microsome fraction. Our study supported this explanation since similar proportions of, for example, ER-related and plasma membrane proteins were found in the discard pellet from the first centrifugation step (7400*g* for 10 min; Figure S1b,e,g) as in the HH and HL. However, it cannot be ruled out that some fraction of the ER-related proteins in the discard pellet derives from residual intact cells, escaping the homogenization.^44,46^

Finally, although “liver microsomes” is a well-established concept, there is a multitude of protocols available for the isolation process with varying number of steps, centrifugation speeds, and times.^8,12,26,43−46,61,62^ These inconsistencies in protocols most certainly contribute to the variable protein levels of CYP and UGT enzymes reported in the HLM fraction.^63,64^ Our study followed the same protocol HLM preparations as several other groups.^26,27,65−69^ We conclude that it is very difficult to completely separate subcellular components during differential centrifugation, and that lack of harmonization of centrifugation protocols contributes to differences between HLM preparations across studies.^15−17^ The incomplete separation of ER-localized drug metabolizing enzymes in the HLM preparations, and the contamination with proteins supposedly localized to other subcellular compartments, point to a need for improving and standardizing HLM preparation protocols while making use of advances in protein quantification.^70^

### Influence of Individual CYP Amount on Drug Metabolic CL_int_

Concentrations of CYP enzymes in the HLM from the 15 donors were comparable with, or higher than, previously reported concentrations in HLM (Figure S2a).^19^ However, the variable degree of enrichment for different enzymes and batches further complicates the use of MPPGL as a scaling factor for substrates that probe specific CYP enzymes. As the metabolic activity should be dependent on the amount of relevant CYP in the incubation, we hypothesized that the AFD and correlations between CL_int_ from HLM and HH would improve by taking into account the amount of specific CYP for each donor of the respective *in vitro* system.

Adjusting for CYP content improved the rank order correlations for three compounds (midazolam, diclofenac, and bufuralol) and remained unchanged at a high correlation level for omeprazole. This indicates that the specific CYP content partially compensated for the inter-individual variability. Surprisingly however, the absolute numerical agreement (AFD) between HH and HLM CL_int_ was only improved by adjusting for the CYP content for midazolam but not the other compounds. The systematically higher unadjusted CL_int_ for midazolam in HLM compared to HH (AFD = 2.95) was in agreement with the 3.5-fold higher levels of the probed CYP3A4 for HLM. This was reflected in the improved AFD (from 2.95 to 1.63) after adjusting the CL_int_ with CYP3A4 amount, instead of mg protein and million hepatocytes. For the other compounds, high CYP enzyme enrichment in the HLM was not reflected in proportionally higher CL_int_ (omeprazole, bufuralol, and bupropion), leading to over-compensation after adjusting for CYP content, and thus AFD was reduced below unity (Table 2; Table S3). In contrast, for diclofenac, the low enrichment of CYP2C9 in HLM led to higher AFD after adjusting for CYP content. A likely explanation is that the proteomics analysis measures the total amount of protein in the two systems; this might not always reflect activity. Thus, overall the compensation for specific CYP content improved correlations but in some cases introduced systematic offsets in CL_int_.

The correlations between bupropion CL_int,mic_ and CYP2B6 concentration (*r* = 0.33) and diclofenac CL_int,mic_ and CYP2C9 (*r* = 0.18), respectively, were lower than those observed earlier in HLM.^71,72^ This might explain why the CL_int_ predictions did not improve as much for these compounds as for midazolam. This contradiction in degree of correlation between CL_int_ and CYP concentration could be because the two previous studies determined the CL_int_ based on formation rates of specific metabolites. In contrast, we used the commonly applied substrate depletion which includes the contribution of all possible metabolic pathways.^33,73,74^ For instance, for both omeprazole and bufuralol, CL_int_ values were relatively high for several donors despite low CYP2C19 or CYP2D6 protein concentrations in HLM and HH. Thus, the limited improvement in AFD after CYP adjustment—for the compounds other than midazolam—could be an effect of that multiple enzymes are involved in the metabolism. This would not be captured in our compensation for only the major CYP probe. For omeprazole, CL_int_ was only compensated for the amount of the main metabolizing enzyme CYP2C19, although this drug is also somewhat metabolized by CYP3A4.^51^ Similarly, bufuralol was only compensated for CYP2D6, while CYP2C19 also, to some extent, contribute to its metabolism.^75^ Likewise, diclofenac was only compensated for CYP2C9, while it is also metabolized by both CYP3A4 and UGT2B7.^76^ By monitoring all formed metabolites (e.g., diclofenac is metabolized to 3-,4-,5-, and acyl glucuronide diclofenac by the three enzymes^77^), the contribution of each enzyme to the drug’s metabolism could be better defined and used to improve the activity–protein concentration correlations. In line with this, the CL_int_ for many of the compounds in this study correlated well with several non-probe CYP enzymes in both HLM and HH (Table S4; Figure S4). This further supports that additional enzymes may be involved in their metabolic clearance.

Intriguingly, other drug metabolizing enzymes were found at high levels in the HLMs, such as the ER-related protein groups, flavine-containing monooxygenases (FMOs) and UGTs, as well as cytosolic enzymes such as aldehyde dehydrogenases (ALDHs) and glutathione *S*-transferases (GSTs) (Figure S3a–d; Supporting Information, Results). Although UGT enzymes were not activated with the cofactor UDPGA in these experiments, both GSTs and FMOs may be active in the HLM under these conditions.^78,79^ Both GSTs and FMOs metabolize a wide selection of different drug compounds.^80−83^ These enzymes could influence the metabolism of drugs in the HLM, and the possible contribution of these enzymes to the drug clearance warrants further investigation. This further complicates the scaling with specific CYP amount in the two systems.

### Compensating the CL_int_ with Intracellular Concentrations

Intracellular unbound drug concentration has previously been used in: bridging differences between biochemical and cellular potency assays (IC_50_);^21^ predicting time-dependent CYP inhibition;^22^ and explaining differences in CYP enzyme inhibition in microsomes and hepatocytes.^23^ We therefore investigated whether Kp_uu_ could also explain the observed system-dependent differences in metabolic CL_int_ of the five substrates. The hypothesis was that active transport and/or metabolic processes in intact hepatocytes could result in non-unity Kp_uu_, that is, that more or less compound is available for metabolism in HH than in HLM. While Kp_uu_ adjustment improved the systematic differences in CYP adjusted CL_int_ in HLM and HH for diclofenac and bufuralol (AFD improving from 1.56 to 1.11 and 0.42 to 1.08), it did not provide a satisfying explanation for the CL_int_ differences between HLM and HH for all five substrates. Previous successful applications of Kp_uu_^21−23^ have assessed the intracellular exposure of molecules which were kept at relatively constant concentrations throughout the assay, for example, enzyme inhibitors or drugs with intracellular targets. In the present study, the constant metabolic removal of drug from the system likely shifted the ratio of intra-to-extracellular concentrations, to an extent depending on the relative rates of membrane passage and metabolic CL_int_. Unknown transport mechanisms could further shift the equilibrium, for example, diclofenac and midazolam may be substrates of efflux transporters.^14,84,85^ To overcome the limitations of membrane passage in hepatocytes, permeabilized hepatocytes might be an alternative for drug metabolism studies.^86^ However, the validity of these results in scaling to *in vivo* remains to be established.

## Conclusions

In this study, we investigated the influence of specific protein amounts and intracellular unbound drug concentration (Kp_uu_) on the CL_int_ of prototypical probe substrates in HLM and HH. To our knowledge, this is the first comparison of drug metabolic activity and global proteomic profiles in HLM and HH using matching donors.

We show systematic differences in the CL_int_ measured in donor-matched HH and HLM, demonstrating that such differences are not merely an effect of inter-individual variability. We outline important differences in the proteomic profiles in HL, HH, and HLM, indicating variable enrichment of supposedly ER-localized CYP enzymes in the preparation of HLM. This suggests that HLM may not equally and accurately capture hepatic metabolic capacity for all CYPs. Contamination of HLM with cytosolic-annotated enzymes is a further complication when using these systems to delineate complex metabolic pathways; it could also be that important drug-metabolizing enzymes are incorrectly annotated.

Together, our findings demonstrate that these factors do not provide a simple one-size-fits-all explanation for differences between HLM and HH. It is possible that these compounds are: metabolized by additional enzymes to the ones probed; that cycling of cellular CYP enzymes between active and inactive states complicates the application of measured protein concentrations; or that the non-steady-state nature of the metabolically competent system and/or saturation of active transport processes confounds the measurement of intracellular drug concentrations. These factors should be evaluated in the future to avoid the use of empirical scaling factors and improve predictions of drug metabolism.

## Supporting Information Available

Supporting Information: Characteristics of the 15 liver donors; LC-MS/MS conditions in the compound quantification; kinetics and proteomics data for the 15 donors; correlation coefficients from CL_int_ of five probe substrates compared with CYP concentrations in HLM and HH from 15 donors; protein expression of CYPs, UGTs, FMOs, and cytosolic enzymes; metabolic activity and protein expression; drug accumulation and unbound fractions of drugs; metabolic clearance of midazolam, omeprazole, bupropion, diclofenac, and bufuralol; and adjusting CL_int,mic_ and CL_int,hep_ with factors influencing metabolic clearance (PDF)
Data S1: Global proteomics data of HH, HLM, HL, and discard pellet (XLSX)
Data S2: Bioinformatics results from global proteomics (XLSX)

## Notes

The mass spectrometry proteomics data have been deposited to the ProteomeXchange Consortium via the PRIDE^87^ partner repository with the data set identifier PXD014131.
