# Supplementary material for: Influence of Proteome Profiles and Intracellular Drug Exposure on Differences in CYP Activity in Donor-Matched Human Liver Microsomes and Hepatocytes
Source: Mol Pharm. 2021 Mar 19;18(4):1792–805. doi: 10.1021/acs.molpharmaceut.1c00053 (PMC8041379; doi:10.1021/acs.molpharmaceut.1c00053)
Supplement: Supplementary file 1 — mp1c00053_si_001.pdf [file mp1c00053_si_001.pdf]

Supplementary Information

Influence of proteome profiles and intracellular drug exposure on differences in CYP activity in donor-matched human liver microsomes and hepatocytes

Christine Wegler[1,2], Pär Matsson[1], Veronica Krogstad[3], Jozef Urdzik[4], Hege Christensen[3], Tommy B. Andersson[2], Per Artursson*[5]

[1]Department of Pharmacy, Uppsala University, Uppsala, Sweden
[2]DMPK, Research and Early Development Cardiovascular, Renal and Metabolism, BioPharmaceuticals R&D, AstraZeneca, Gothenburg, Sweden
[3]Department of Pharmaceutical Biosciences, School of Pharmacy, University of Oslo, Oslo, Norway
[4]Department of Surgical Sciences, Uppsala University, Uppsala, Sweden
[5]Department of Pharmacy and Science for Life Laboratory, Uppsala University, Uppsala, Sweden

Supplementary Results

Protein profiling of human liver microsomes, hepatocytes, and homogenates

Principal component analysis (PCA), including proteins that were quantified with at least three peptides, showed that the HLM differed from both the HL and HH along the most influential first principal component axis (PC1 46.2%; Figure 2a). Analysis of protein function in the proteomes of the three sample types, using Proteomaps [1], showed that biosynthesis processes such as amino acid metabolism, glycolysis, carbohydrate metabolism, and lipid metabolism dominated in all three sample types (Figure 2b). Additionally, the HLM Proteomap displayed a larger content of proteins related to the endoplasmic reticulum (ER) as compared to those from the HL and HH (Figure 2b).

Further, the majority of the proteins quantified with at least three unique peptides across the HL, HH, and HLM (3075 of 3989) were found in all three sample types (Figure 2c). Despite the large overlap in number of quantified proteins, the protein levels in the HLM differed from both the HL and HH with absolute average fold differences (AAFD) of 3.0 and 3.1, respectively (Figure 2d). As indicated from the Proteomaps, HLM proteins with significantly higher concentrations (Figure S1a; Volcano-plot: FDR = 0.01, S0 = 2) were involved in pathways associated with the ER, such as fatty acid and drug metabolism (Data S2). This is in line with that HLM are suggested to be vesicles derived from the ER [2].

An in-depth investigation of the fractional contribution (total protein content %) of proteins from different subcellular locations [3] confirmed that ER-annotated proteins made up a larger proportion of the total protein content in the HLM (19%) than those in the HL (7%) and HH (9%; Figure 2e). However, the HLM also contained large proportions of proteins associated with other subcellular compartments, including mitochondria (14% of the total HLM protein content, compared to 17% and 21% in HL and HH, respectively). Furthermore, the HLM contained a comparable proportion of cytosolic proteins (27%) as to those in the HL (27%) and HH (32%). This was surprising since the cytosolic proteins are assumed to be discarded during the HLM preparation [4]. The HLM also contained a large proportion of nuclear proteins (19%) that are expected to be captured in the normally discarded pellet obtained in the first low-speed centrifugation step [4-7] (Figure 2e). The fraction of nuclear proteins in HLM were comparable to that in the discard pellets (20%), and not much lower than either HL (28%) or HH (24%; Figure S1b-d). This demonstrates that the HLM fractions are “contaminated” with many proteins that are not associated with the ER compartment, an observation that is supported by previous investigations [8, 9].

Similar to what was observed for the complete set of quantified proteins, most of the proteins classified as ER-localized were found at substantial concentrations in the three sample types (HL, HH, and HLM) and the discard pellet (Figure S1e-h). The ER-related proteins were in general enriched 2.8-fold (Figure 2f) in the HLM with a large variability in enrichment ranging from 0.03–120-fold across the different proteins. Since ER-associated proteins constituted 7% of the total protein content in our HL, assuming complete isolation and full recovery of the ER-fraction in the HLM, we would expect a 14-fold enrichment of microsomal ER proteins. The lower enrichment of ER-proteins was also reflected in the 2.2 to 4.6-fold enrichment of the ER-membrane markers, CANX and POR, which were similar or slightly lower than previous estimates (Figure S1i) [10]. The traditional ER-activity markers glucose-6-phosphatase (G6PC) and HMG-CoA reductase (HMGCR)[11], were also enriched to the same extent (3.4 to 7.4-fold) in the HLM (Figure 2g). Furthermore, we found specific membrane markers for other organelles, e.g., lysosome, peroxisome, and plasma membrane, in the HLM, where the enrichment were in agreement with previous findings (Figure S1i) [10]. The variability in enrichment of different ER-related proteins (0.03 to 120-fold) demonstrates the complexity of the enrichment process, which limits the use of specific protein markers as scaling factors for recovery of ER-related proteins.

Enrichment of CYP enzymes and the effect on metabolic clearance

The isolation of ER-localized proteins in liver HLM should lead to enhanced levels of membrane bound drug metabolizing enzymes, such as CYPs [12]. The median concentrations of the CYP enzymes in the HLM ranged from 1.3 (CYP2J2) to 76.5 (CYP2C8) fmol/µg protein, and are comparable or higher than previously reported concentrations in HLM (Figure S2a) [9]. The CYP enzymes in HLM were enriched

on average 3.2-fold (range 1.2 to 56) compared to CYP levels in the HL and HH (HL and HH CYP levels that were in good agreement with that previously reported; Figure S2b-c [13-15]). Interestingly, two of the enzymes from the subfamily CYP2C (CYP2C9 and CYP2C19) were very differently enriched in the HLM. CYP2C9, with higher HL concentrations, was poorly enriched (AFD 1.4-fold) in the HLM, while CYP2C19, with lower HL concentrations, had approximately 50-fold higher concentration in the HLM compared to both HL (56-fold) and HH (46-fold) (Figure 2a). In line with this, global analysis showed that proteins with higher initial HL concentrations were less enriched in the HLM compared to those with lower concentrations (r_s = –0.33; Figure S3e), suggesting a saturation in the enrichment process.

Since the liver HL, HH, and HLM were obtained from 15 matched donors, we were also able to investigate the preservation of the relative expression of the different enzymes across the donors from the three sample types. In general, the donor rank orders for the different CYPs were highly correlated with median Spearman's correlations (r_s) of 0.87 (Figure 3a; significant Spearman's rank correlations > 0.7, $p < 0.006$, after Bonferroni correction for multiple comparisons) across the three sample types (range across all CYPs and sample types r_s = 0.01 to 0.99). However, some enzymes of great importance for drug metabolism, including CYP3A4 and CYP2C19, displayed weaker rank correlations between HLM and HH (r_s = 0.48 and $r_{s\,=}$ 0.66, respectively), which could give rise to differences in metabolic clearance.

Enrichment of other drug metabolizing enzymes in human liver microsomes

The UGT enzymes were in general not as highly enriched in the HLM as the CYP enzymes, with respective median fold-enrichments of 2.6 (range: 1.3-4.3) and 2.4-fold (range: 0.5-5.8) compared to the levels in the HL and HH. The inter-donor spread of the UGT enzymes were similar in all three sample types with median values of 3 (range: 2-14) in HL, 3 (range: 2-28) in HH, and 3 (range: 2-17) in the HLM. Further, the rank order correlations across the 15 donors were not as well preserved for the UGT enzymes as for the CYP enzymes. The median Spearman's rank correlations were 0.76 (range r_s: 0.45-0.81) between HLM and HL, 0.52 (range r_s: 0.10-0.83) between HLM and HH, and 0.77 (range r_s: 0.17-0.99) between HH and HH (Figure S3a).

The FMO enzymes were enriched to the same extent as the CYP enzymes in the HLM, with median fold-enrichments of 3.4 (range: 2.7-3.5) and 2.8 (range: 1.8-3.1) compared to HL and HH, respectively. High rank order correlations were obtained for the FMO enzymes with median Spearman's correlations of 0.93 (range r_s: 0.86-0.94) between HLM and HL, 0.91 (range r_s: 0.81-0.91) between HLM and HH, and 0.85 (range r_s: 0.81-0.84) between HL and HH. Notably, FMO1 was only found in the HLM at low concentrations ranging from 0.004 to 0.2 fmol/µg protein (quantified with on average four unique peptides; Figure S3b).

Additionally, many cytosolic enzymes involved in drug metabolism, such as aldehyde dehydrogenases (ALDHs), sulfotransferases (SULTs), and glutathione S-transferases (GSTs) were also found in the HLMs (Figure S3c, S3d). Although the concentrations were on average lower in the HLM, where the geometric mean was 5.9 fmol/µg protein (range across enzymes 0.01 – 257.3) as compared to HL (geometric mean: 11.4; range: 0.02 – 312.8) and HH (geometric mean: 10.7; range 0.01 – 380.3) fmol/µg protein, several of the proteins were still quantified at high levels in the HLM. For instance, the phase I and II metabolizing enzymes, ALDH1A1 and GSTA2, respectively, had geometrical mean concentrations of 41.4 and 153.1 fmol/µg protein in the HLMs.

Supplementary Tables and Figures

Table S1. Characteristics of the 15 liver donors

Age (mean)	64 (39 - 79)[a]
BMI (mean, kg/m^2)	25.7 (20.1 - 32.9)[a]
Gender	
Male	10
Female	5
Diagnosis[b]	
CRC	13
BC	1
GIST	1

[a]Minimum and maximum, [b]CRC: colorectal cancer, BC: breast cancer, GIST: Gastrointestinal stromal tumor

Table S2. LC-MS/MS conditions in the compound quantification.

Compound	Injection volume (μL)	Retention time (min)	MS method				
			Parent m/z	Cone Voltage	Daughter m/z	Collision Energy	Ionization
Midazolam	5	0.98	326.0	44	291.0	28	ESI+
Omeprazole	5	0.91	346.2	16	198.1	12	ESI+
Bufuralol	5	0.98	262.0	16	160.9	25	ESI+
Bupropion	5	0.94	241.0	16	131.9	25	ESI+
Diclofenac	5	1.24	294.0	18	250.0	15	ESI-
1-OH-Midazolam	5	0.95	342.1	34	203.0	28	ESI+
4-OH-Midazolam	5	0.95	342.1	36	234.0	22	ESI+
OH-Bupropion	5	0.89	256.9	16	131.9	28	ESI+
4-OH-Diclofenac	5	1.11	312.1	22	230.1	32	ESI+
Warfarin (I.S.)	--	1.19	309.2	22	163.0	14	ESI+
			307.2	40	161.0	22	ESI-

Table S3. Kinetics and proteomics data for the 15 donors

Midazolam

	CYP3A4 (pmol/mg protein)			pmol CYP3A4 in incubation		Kp_{uu}	$CL_{int,mic}$ (ml/min/kg bw)			$CL_{int,hep}$ (ml/min/kg bw)	
Batch	HLM	HH	HL	HLM	HH	HH	eq. 7	eq. 8	eq. 9	eq. 7	eq.8
UU1	94.2	25.8	29.4	23.6	17.5	0.39	466.2	297.3	117.1	168.4	221.9
UU2	58.3	19.5	15.0	14.6	18.1	0.15	265.7	139.5	20.6	86.4	44.8
UU3	75.0	32.2	19.5	18.8	23.9	0.31	366.7	194.5	60.5	192.0	116.3
UU4	81.8	14.5	17.7	20.4	11.8	0.23	516.4	229.1	52.7	98.9	121.6
UU5	91.1	27.6	21.2	22.8	19.7	0.37	736.2	349.8	129.6	220.3	151.3
UU6	45.9	24.0	12.5	11.5	9.8	0.55	374.7	209.2	115.7	145.8	172.8
UU7	48.6	18.6	12.5	12.2	19.4	0.50	318.9	167.0	83.7	126.7	81.6
UU8	136.6	14.7	28.5	34.2	9.1	0.67	818.9	349.1	232.7	105.2	356.3
UU9	37.9	8.8	9.0	9.5	7.9	0.30	203.4	98.5	29.2	45.5	41.6
UU10	72.4	21.3	18.9	18.1	17.1	0.21	423.1	226.2	47.0	128.2	111.0
UU11	90.3	30.0	20.3	22.6	22.7	0.29	352.5	162.0	47.5	170.5	131.4
UU12	73.4	23.9	16.6	18.3	16.6	0.32	377.9	174.9	55.1	134.0	98.3
UU13	112.2	54.6	28.0	28.0	37.4	0.21	757.7	387.2	81.5	413.1	213.3
UU14	87.7	22.4	20.5	21.9	17.8	0.29	356.9	170.7	49.7	225.8	184.7
UU15	52.1	8.6	14.1	13.0	6.0	0.38	236.3	130.5	49.5	56.4	126.0

Omeprazole

	CYP2C19 (pmol/mg protein)			pmol CYP2C19 in incubation		Kp_{uu}	$CL_{int,mic}$ (ml/min/kg bw)			$CL_{int,hep}$ (ml/min/kg bw)	
Batch	HLM	HH	HL	HLM	HH	HH	eq. 7	eq. 8	eq. 9	eq. 7	eq.8
UU1	64.7	1.9	2.1	16.2	1.3	1.23	39.8	2.7	3.3	21.4	27.7
UU2	50.9	0.2	0.1	12.7	0.2	1.76	1.7	0.0	0.0	4.5	2.9
UU3	60.4	5.5	3.6	15.1	4.1	1.43	72.8	8.8	12.6	79.2	47.3
UU4	52.0	2.8	2.5	13.0	2.3	1.11	63.9	6.2	6.9	32.9	23.9
UU5	87.8	6.0	4.8	22.0	4.3	0.96	109.2	12.3	11.8	55.3	38.6
UU6	70.2	4.5	1.9	17.6	1.8	0.71	40.5	2.3	1.6	16.6	11.1
UU7	39.2	0.7	0.6	9.8	0.7	1.98	28.2	0.8	1.6	10.8	7.4
UU8	59.1	0.6	0.7	14.8	0.3	1.21	52.6	1.3	1.6	8.7	9.7
UU9	47.4	0.3	0.3	11.9	0.3	1.49	13.6	0.2	0.3	4.6	4.2
UU10	45.6	0.1	0.1	11.4	0.0	1.23	18.3	0.0	0.1	5.7	5.3
UU11	69.9	5.9	4.9	17.5	3.9	1.51	88.0	12.5	18.9	144.4	118.7
UU12	38.9	1.2	0.8	9.7	0.9	1.90	33.1	1.4	2.6	23.3	16.0
UU13	32.8	1.5	0.5	8.2	1.1	1.08	74.4	2.2	2.4	26.1	10.0
UU14	48.0	1.8	1.2	12.0	1.4	2.62	21.2	1.0	2.7	16.1	10.9
UU15	43.7	0.4	1.1	10.9	0.3	1.79	16.7	0.9	1.5	5.3	11.5

Table S3. Kinetics and proteomics data for the 15 donors cont.

Diclofenac

Batch	CYP2C9 (pmol/mg protein) HLM	CYP2C9 (pmol/mg protein) HH	CYP2C9 (pmol/mg protein) HL	pmol CYP2C9 in incubation HLM	pmol CYP2C9 in incubation HH	Kp_{uu} HH	$CL_{int,mic}$ (ml/min/kg bw) eq. 7	$CL_{int,mic}$ (ml/min/kg bw) eq. 8	$CL_{int,mic}$ (ml/min/kg bw) eq. 9	$CL_{int,hep}$ (ml/min/kg bw) eq. 7	$CL_{int,hep}$ (ml/min/kg bw) eq.8
UU1	46.8	43.9	33.3	11.7	29.8	0.77	494.1	719.8	601.6	547.3	498.4
UU2	39.5	36.7	29.3	9.9	34.1	0.36	265.6	402.4	314.8	636.7	342.2
UU3	26.2	26.4	20.5	6.6	19.6	0.48	671.2	1070.7	597.3	385.0	299.0
UU4	26.4	21.1	19.6	6.6	17.2	0.35	701.9	1063.1	454.8	332.5	310.5
UU5	44.6	32.3	30.7	11.2	23.1	0.33	640.3	902.2	287.1	392.3	333.5
UU6	40.2	40.1	33.8	10.1	16.3	1.16	549.3	944.8	1050.6	400.6	768.3
UU7	29.3	21.8	20.6	7.3	22.8	0.96	353.2	507.4	483.8	263.4	238.4
UU8	42.7	26.5	28.3	10.7	16.4	2.14	617.5	837.0	1737.0	322.4	600.8
UU9	32.2	25.1	16.6	8.0	22.4	0.49	628.1	662.9	388.0	361.8	216.1
UU10	57.3	46.6	42.3	14.3	37.4	1.02	782.0	1182.2	857.8	608.7	538.4
UU11	35.4	25.8	21.2	8.9	18.6	0.43	444.8	543.6	409.5	486.6	434.9
UU12	27.4	18.9	13.2	6.8	13.2	0.49	253.7	250.5	155.4	322.6	237.3
UU13	27.1	27.4	18.9	6.8	18.8	0.66	279.1	397.7	492.5	369.1	255.5
UU14	38.3	33.4	21.5	9.6	26.4	0.25	211.6	242.4	96.0	448.4	258.0
UU15	37.6	29.8	28.4	9.4	20.5	0.39	128.6	198.6	125.8	584.8	713.5

Bupropion

Batch	CYP2B6 (pmol/mg protein) HLM	CYP2B6 (pmol/mg protein) HH	CYP2B6 (pmol/mg protein) HL	pmol CYP2B6 in incubation HLM	pmol CYP2B6 in incubation HH	Kp_{uu} HH	$CL_{int,mic}$ (ml/min/kg bw) eq. 7	$CL_{int,mic}$ (ml/min/kg bw) eq. 8	$CL_{int,mic}$ (ml/min/kg bw) eq. 9	$CL_{int,hep}$ (ml/min/kg bw) eq. 7	$CL_{int,hep}$ (ml/min/kg bw) eq.8
UU1	7.7	2.5	2.1	1.9	1.7	2.10	27.5	15.1	31.7	79.7	77.1
UU2	4.1	1.4	0.9	1.0	1.3	1.27	23.3	9.9	12.6	47.7	20.3
UU3	3.6	1.2	0.8	0.9	0.9	1.24	38.0	17.2	21.3	58.4	40.4
UU4	12.8	4.4	2.9	3.2	3.6	1.55	33.3	15.2	23.5	106.5	70.0
UU5	31.2	8.3	5.6	7.8	5.9	0.89	62.4	23.1	20.5	90.8	55.5
UU6	2.1	0.8	0.7	0.5	0.3	0.46	27.1	17.4	8.1	41.0	79.8
UU7	3.8	1.2	1.0	1.0	1.2	2.11	10.9	5.9	12.5	87.9	72.4
UU8	8.4	1.9	1.7	2.1	1.2	2.96	14.1	5.7	16.8	60.2	89.9
UU9	4.7	1.5	1.0	1.2	1.3	1.79	22.2	9.9	17.6	87.8	55.2
UU10	6.2	2.2	2.2	1.5	1.8	0.84	20.7	15.0	12.7	61.5	58.6
UU11	8.1	2.0	2.0	2.0	0.8	0.90	24.5	12.4	11.1	47.5	54.5
UU12	7.4	1.8	1.5	1.8	1.3	1.17	60.4	25.8	30.3	62.1	56.2
UU13	4.4	1.4	1.0	1.1	0.9	0.49	38.4	18.4	9.0	117.9	88.7
UU14	5.1	1.6	1.0	1.3	1.3	1.58	7.5	3.1	5.0	90.7	52.9
UU15	8.0	3.3	2.3	2.0	2.3	1.14	12.4	7.3	8.4	68.9	66.6

Table S3. Kinetics and proteomics data for the 15 donors cont.

Bufuralol

	CYP2D6 (pmol/mg protein)			pmol CYP2D6 in incubation		Kp_{uu}	$CL_{int,mic}$ (ml/min/kg bw)			$CL_{int,hep}$ (ml/min/kg bw)	
Batch	HLM	HH	HL	HLM	HH	HH	eq. 7	eq. 8	eq. 9	eq. 7	eq.8
UU1	50.9	21.3	16.9	12.7	14.4	5.55	70.4	47.8	265.5	146.1	134.4
UU2	30.1	12.3	8.1	7.5	11.4	1.41	22.6	12.4	17.6	57.7	25.7
UU3	0.1	0.1	0.4	0.0	0.1	3.14	70.5	438.0	1377.1	64.2	176.4
UU4	12.6	4.3	3.1	3.2	3.5	2.35	35.4	17.9	42.0	52.8	38.3
UU5	0.2	0.1	0.1	0.1	0.0	2.76	21.5	26.9	74.3	74.5	126.2
UU6	58.7	27.4	20.3	14.7	11.2	2.95	50.3	35.7	105.2	65.7	110.6
UU7	56.1	20.2	14.4	14.0	21.1	2.71	52.6	27.6	74.9	107.9	74.1
UU8	21.5	7.0	5.3	5.4	4.3	7.28	36.8	18.4	134.3	35.0	46.2
UU9	34.2	10.5	7.1	8.6	9.4	3.11	37.0	15.7	48.7	70.9	43.2
UU10	41.6	15.6	12.8	10.4	12.5	1.50	59.0	37.1	55.6	83.5	66.6
UU11	23.8	4.5	6.1	5.9	0.1	0.80	28.2	14.7	11.8	53.1	82.0
UU12	18.7	5.8	4.8	4.7	4.0	3.62	20.3	10.7	38.7	54.8	48.2
UU13	57.8	23.5	16.6	14.4	16.1	1.82	64.6	37.9	69.1	77.3	54.9
UU14	0.1	0.1	0.2	0.0	0.1	3.25	ND	ND	ND	94.4	169.8
UU15	3.8	1.4	1.3	0.9	1.0	1.66	ND	ND	ND	22.0	26.1

Table S4. Correlation coefficients from CL_{int} of five probe substrates compared with CYP concentrations in HLM and HH from 15 donors

		Bufuralol		Bupropion		Diclofenac		Midazolam		Omeprazole	
		HH	HLM	HH	HLM	HH	HLM	HH	HLM	HH	HLM
CYP1A2	r	0.49	-0.06	0.09	0.29	-0.54	0.33	0.62	0.53	0.52	0.64
	r_s	0.30	-0.13	0.09	0.38	-0.49	0.17	0.55	0.46	0.53	0.59
CYP2B6	r	-0.18	-0.49	0.40	0.33	0.08	0.15	-0.04	0.45	0.20	0.37
	r_s	-0.20	-0.49	0.37	0.15	0.15	0.15	-0.09	0.41	0.15	0.33
CYP2C19	r	0.12	-0.20	0.04	0.25	-0.33	0.51	0.54	0.21	0.88	0.32
	r_s	0.02	-0.10	0.05	0.25	-0.21	0.44	0.66	0.19	0.90	0.47
CYP2C8	r	-0.23	-0.73	-0.20	0.32	0.09	0.23	-0.34	0.21	0.02	-0.01
	r_s	-0.27	-0.73	-0.32	0.25	0.13	0.24	-0.14	0.20	-0.05	0.08
CYP2C9	r	0.31	0.01	-0.29	-0.24	0.73	0.18	0.13	0.11	-0.26	-0.22
	r_s	0.40	-0.05	-0.23	-0.25	0.75	0.11	0.21	0.18	-0.29	-0.19
CYP2D6	r	0.13	0.14	-0.16	0.00	0.06	0.07	-0.22	-0.06	-0.41	-0.23
	r_s	0.36	0.41	-0.17	-0.02	0.15	0.04	-0.11	0.05	-0.30	-0.11
CYP3A4	r	0.47	0.02	0.01	0.17	0.12	0.13	0.94	0.84	0.64	0.51
	r_s	0.34	0.05	-0.09	0.32	0.18	0.06	0.89	0.76	0.73	0.65
CYP3A5	r	0.26	-0.44	0.24	-0.02	-0.13	-0.09	0.65	0.37	0.41	0.28
	r_s	0.12	-0.27	0.13	0.31	-0.24	0.01	0.79	0.50	0.55	0.58

r = Pearson's correlation coefficient calculated from log-transformed values, r_s = Spearman's rank correlation coefficient

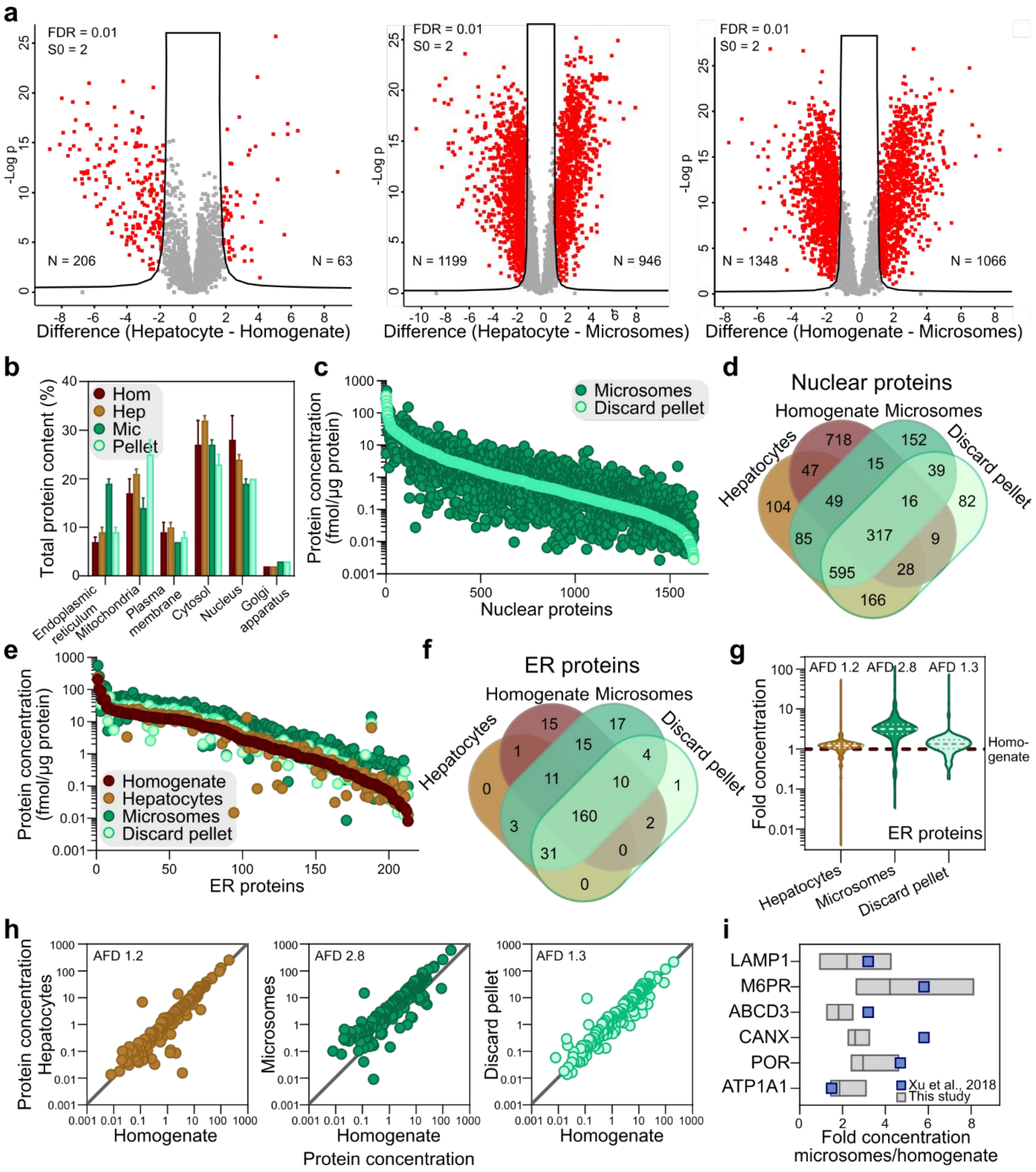

Figure S1. Global proteomics a) Fold difference of protein expression between different sample types (human liver microsomes, hepatocytes, and homogenates), against P-values, displayed in Volcano plots. Red circles mark proteins with significantly different expression. N denotes number of proteins with significantly different expression. b) Total protein content (%) of proteins from different subcellular compartments in human liver microsomes, hepatocytes, homogenates, and first low-speed centrifugation pellets. c) Protein concentrations of nuclear proteins in human liver microsomes and first low-speed centrifugation pellet, sorted by concentrations in the pellet. d) Number and overlap of nuclear proteins in (c). e) Protein concentrations of ER-related proteins in human liver microsomes, hepatocytes, homogenates, and first low-speed centrifugation pellets, sorted by concentrations in the homogenates. f) Number of ER-related proteins in (e). g) Enrichment of ER-related proteins in human liver microsomes, hepatocytes, and first low-speed centrifugation pellets, compared to in the liver homogenates. h) Comparison of median protein concentrations of hepatocytes, microsomes, and discard pellets against homogenate samples. i) Enrichment of membrane markers in the 15 microsomal samples against homogenate, compared with microsomal enrichment in the literature[10]. Gray floating bars denote range, and the line denotes median across the 15 donors.

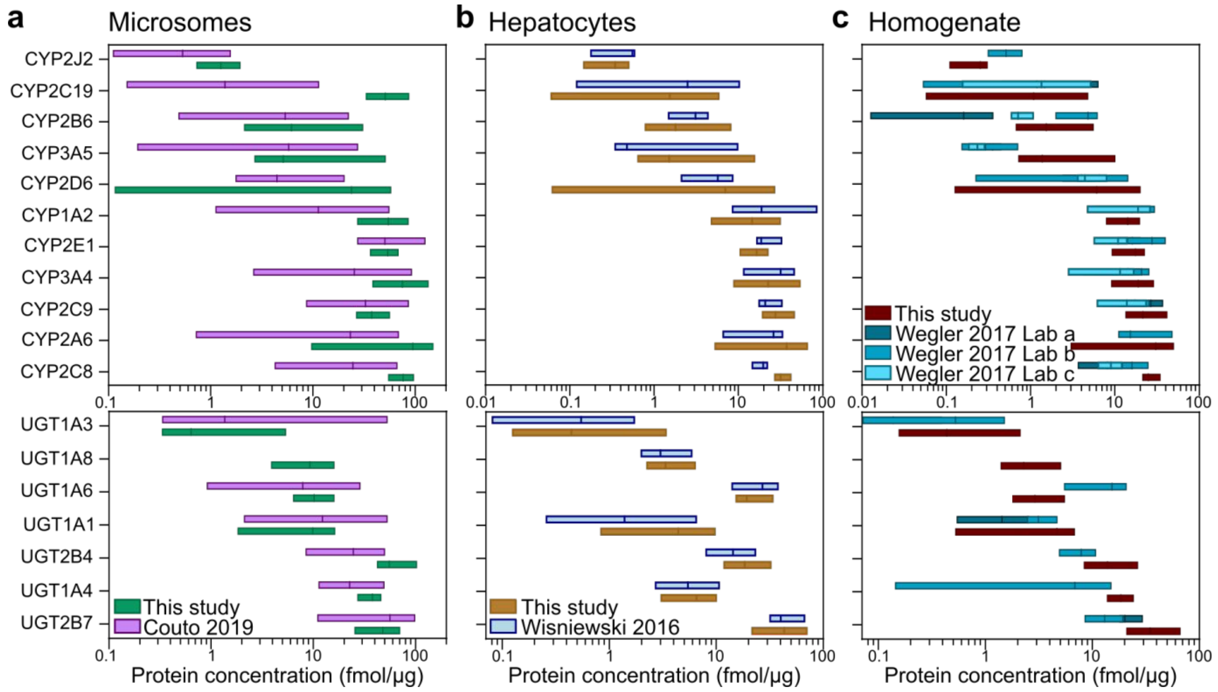

Figure S2. Protein expression of CYPs and UGTs in the a) 15 microsomal samples, compared with literature values [9]; b) 15 hepatocyte samples, compared with literature values [14]; and c) in the 15 homogenate samples, compared with literature values [13]. Floating bars denote range, and the line denotes median across the different samples.

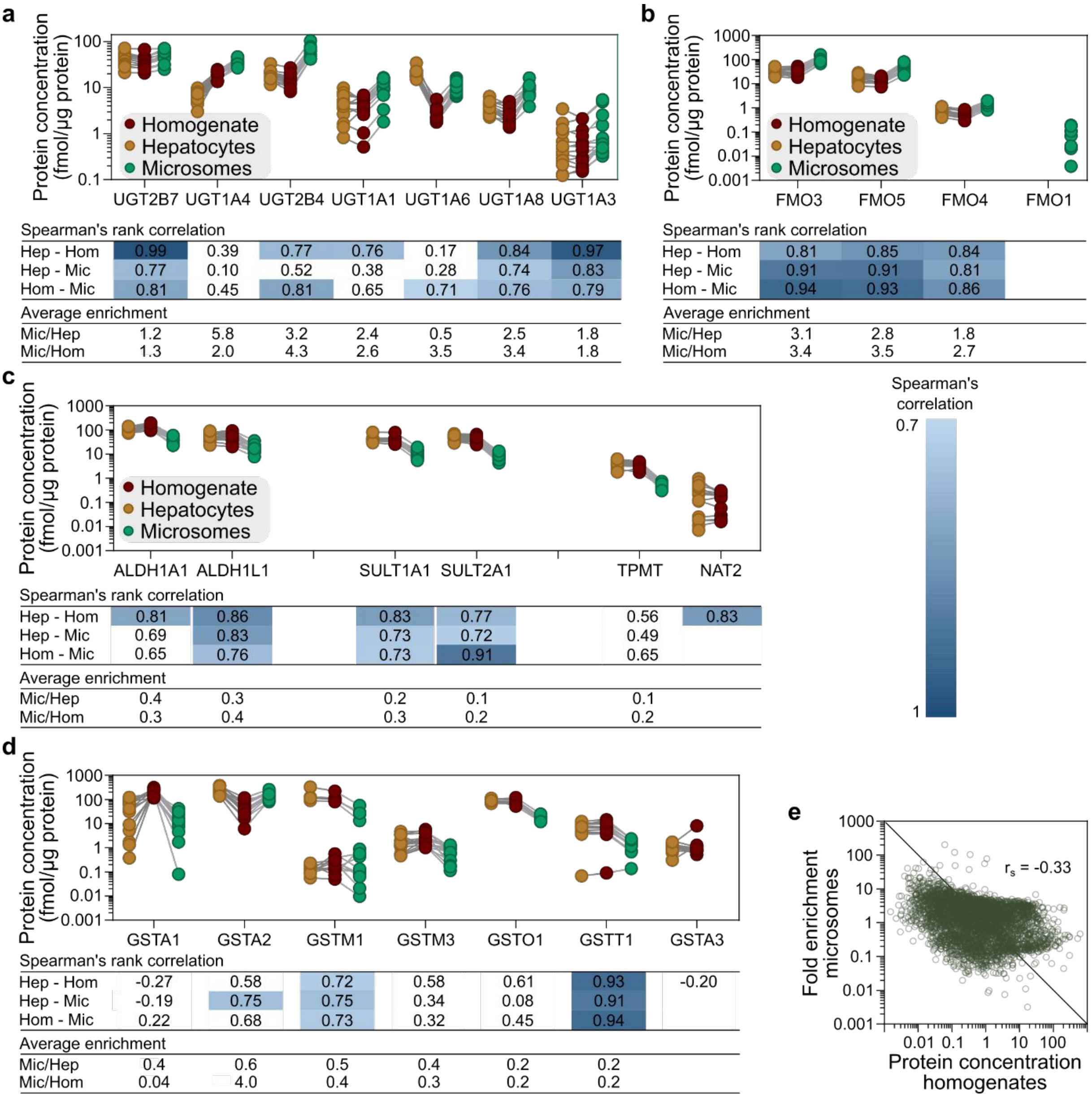

a Spearman's rank correlation / Average enrichment

	UGT2B7	UGT1A4	UGT2B4	UGT1A1	UGT1A6	UGT1A8	UGT1A3
Hep - Hom	0.99	0.39	0.77	0.76	0.17	0.84	0.97
Hep - Mic	0.77	0.10	0.52	0.38	0.28	0.74	0.83
Hom - Mic	0.81	0.45	0.81	0.65	0.71	0.76	0.79
Mic/Hep	1.2	5.8	3.2	2.4	0.5	2.5	1.8
Mic/Hom	1.3	2.0	4.3	2.6	3.5	3.4	1.8

b Spearman's rank correlation / Average enrichment

	FMO3	FMO5	FMO4	FMO1
Hep - Hom	0.81	0.85	0.84	
Hep - Mic	0.91	0.91	0.81	
Hom - Mic	0.94	0.93	0.86	
Mic/Hep	3.1	2.8	1.8	
Mic/Hom	3.4	3.5	2.7	

c Spearman's rank correlation / Average enrichment

	ALDH1A1	ALDH1L1	SULT1A1	SULT2A1	TPMT	NAT2
Hep - Hom	0.81	0.86	0.83	0.77	0.56	0.83
Hep - Mic	0.69	0.83	0.73	0.72	0.49	
Hom - Mic	0.65	0.76	0.73	0.91	0.65	
Mic/Hep	0.4	0.3	0.2	0.1	0.1	
Mic/Hom	0.3	0.4	0.3	0.2	0.2	

d Spearman's rank correlation / Average enrichment

	GSTA1	GSTA2	GSTM1	GSTM3	GSTO1	GSTT1	GSTA3
Hep - Hom	-0.27	0.58	0.72	0.58	0.61	0.93	-0.20
Hep - Mic	-0.19	0.75	0.75	0.34	0.08	0.91	
Hom - Mic	0.22	0.68	0.73	0.32	0.45	0.94	
Mic/Hep	0.4	0.6	0.5	0.4	0.2	0.2	
Mic/Hom	0.04	4.0	0.4	0.3	0.2	0.2	

Figure S3. Protein expression of UGTs, FMOs, and cytosolic enzymes in liver homogenates, isolated hepatocytes, and liver microsomes from the 15 donors. a) UGT b) FMO c) cytosolic enzymes d) glutathione transferase. e) Fold enrichment of proteins in microsomes correlated to initial protein concentration in homogenates. Concentration levels are given in fmol/µg total protein in the respective system. Spearman's rank correlations compare the relative expression of each enzyme across the 15 donors between the respective sample type, where significant correlation coefficients are > 0.7 ($P < 0.006$, after Bonferroni correction for multiple comparisons). Average enrichment of microsomes compared to homogenates and hepatocytes was calculated based on concentrations from the 15 donors.

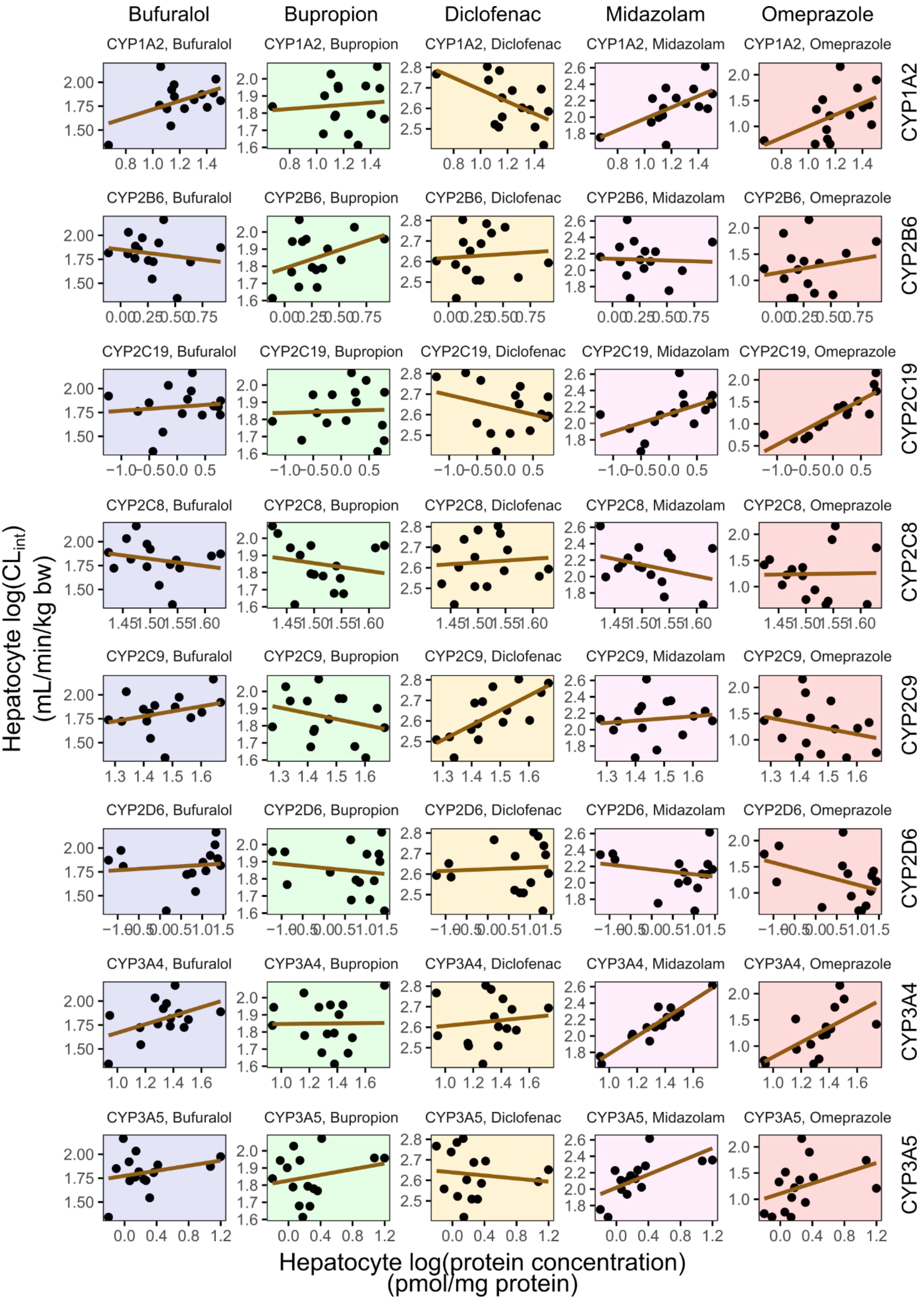

Figure S4a. Metabolic activity and protein expression. Correlations between CL_{int} (eq. 7) in HH of bufuralol, bupropion, diclofenac, midazolam, and omeprazole with CYP concentrations in the HH from 15 donors.

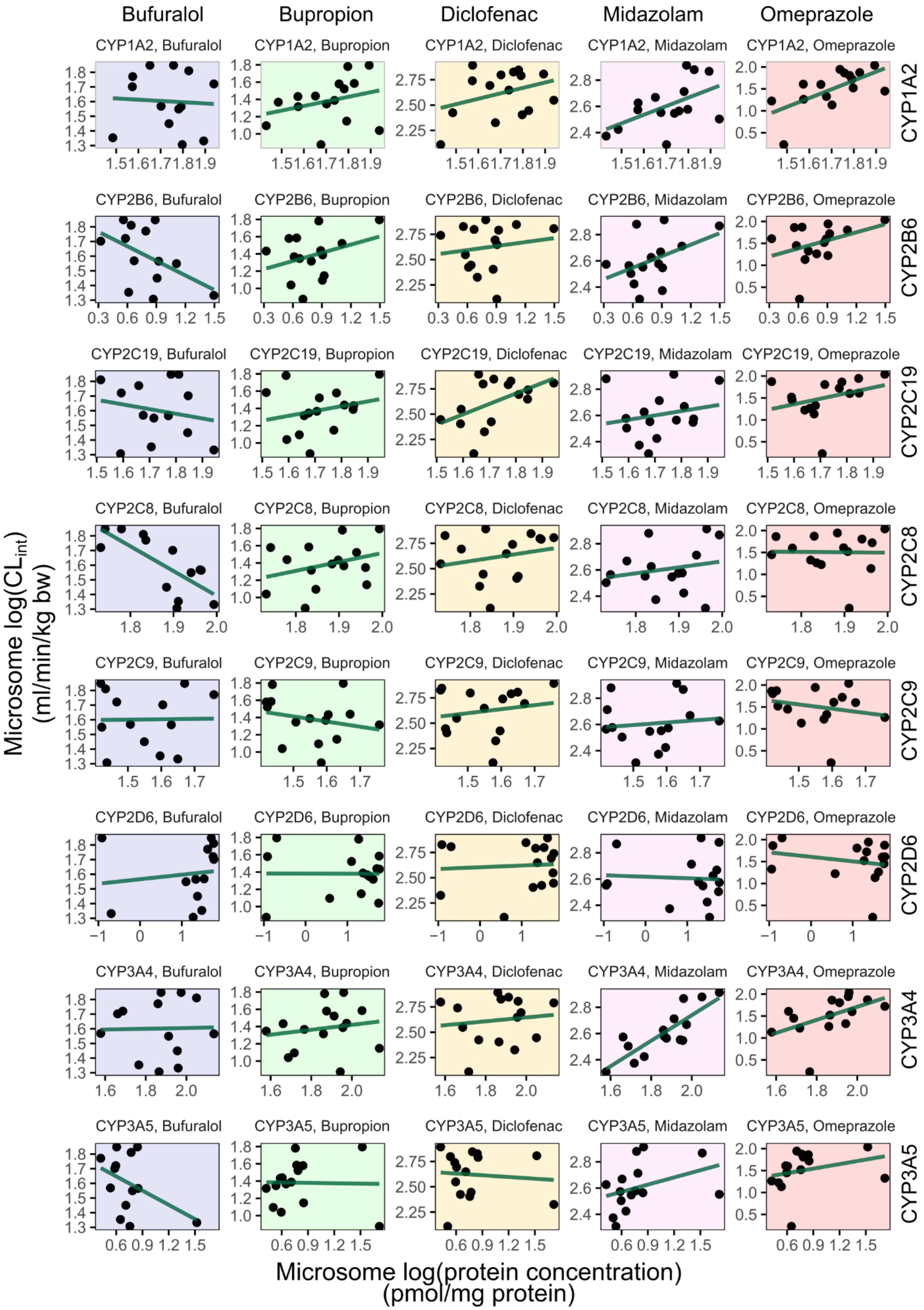

Figure S4b. Metabolic activity and protein expression. Correlations between CL_{int} (eq. 7) in HLM of bufuralol, bupropion, diclofenac, midazolam, and omeprazole with CYP concentrations in HLM from 15 donors.

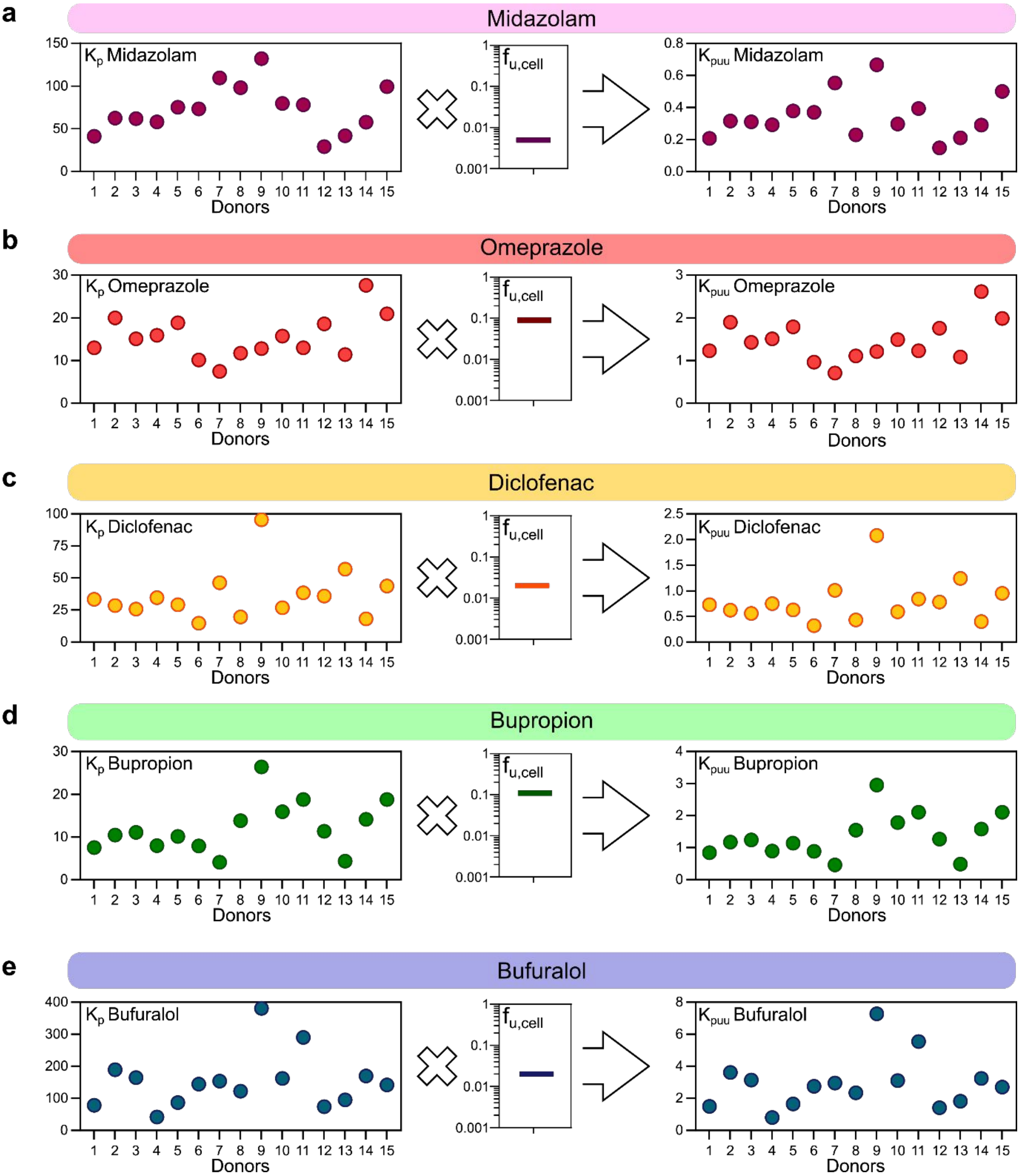

Figure S5. Drug accumulation and unbound fractions of drugs. Drug accumulation (Kp), fraction unbound ($f_{u,cell}$), and corresponding intracellular unbound drug concentration (Kp_{uu}) of a) midazolam, b) omeprazole, c) diclofenac, d) bupropion, and e) bufuralol in human hepatocytes from 15 donors.

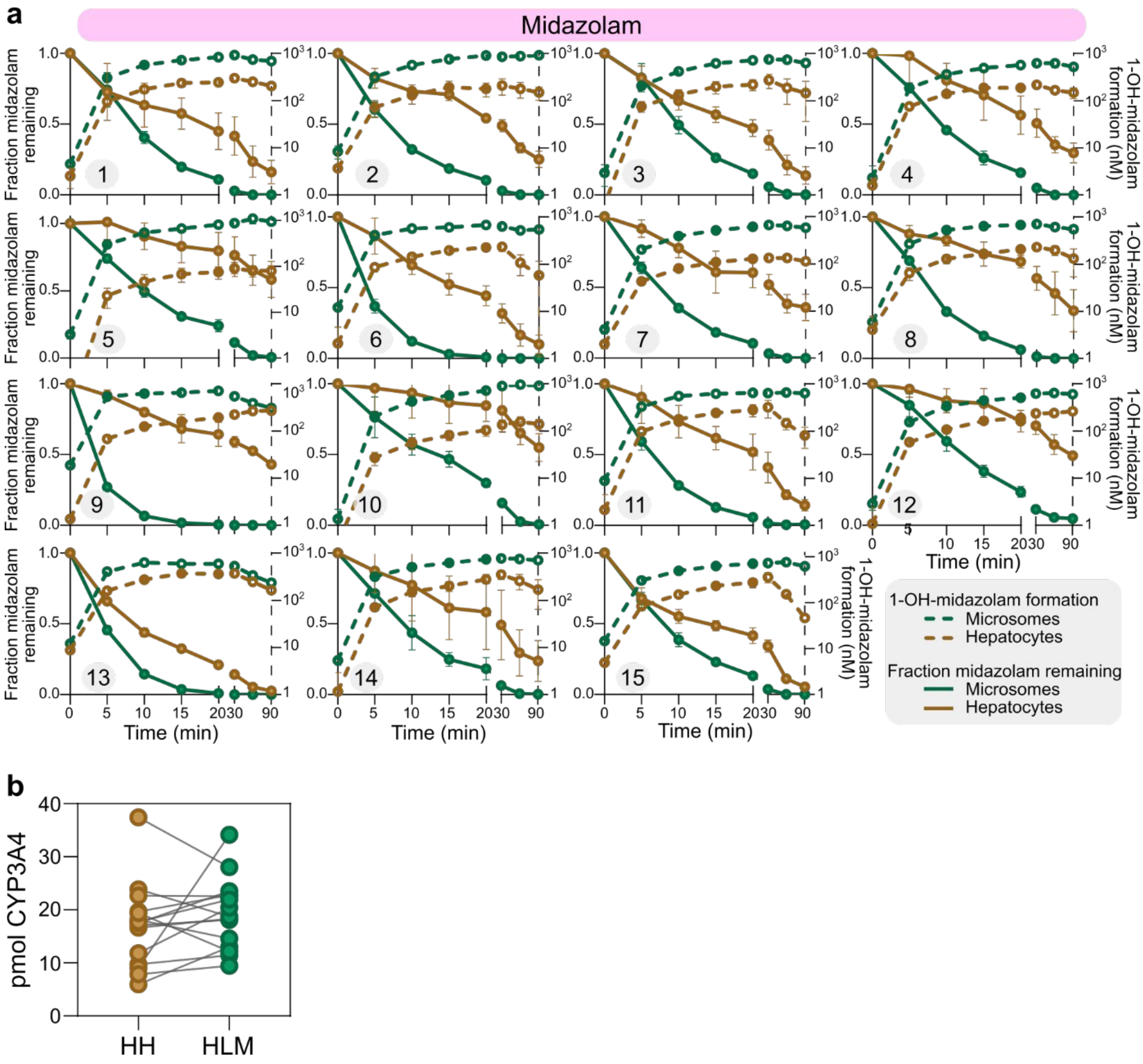

Figure S6. Metabolic clearance of midazolam a) Metabolic clearance of midazolam, measured by depletion of midazolam (fraction remaining) and 1-OH-midazolam formation in HLM and HH from 15 matching donors. b) Amount of CYP3A4 in the incubations of the 15 donors in HLM and HH, respectively.

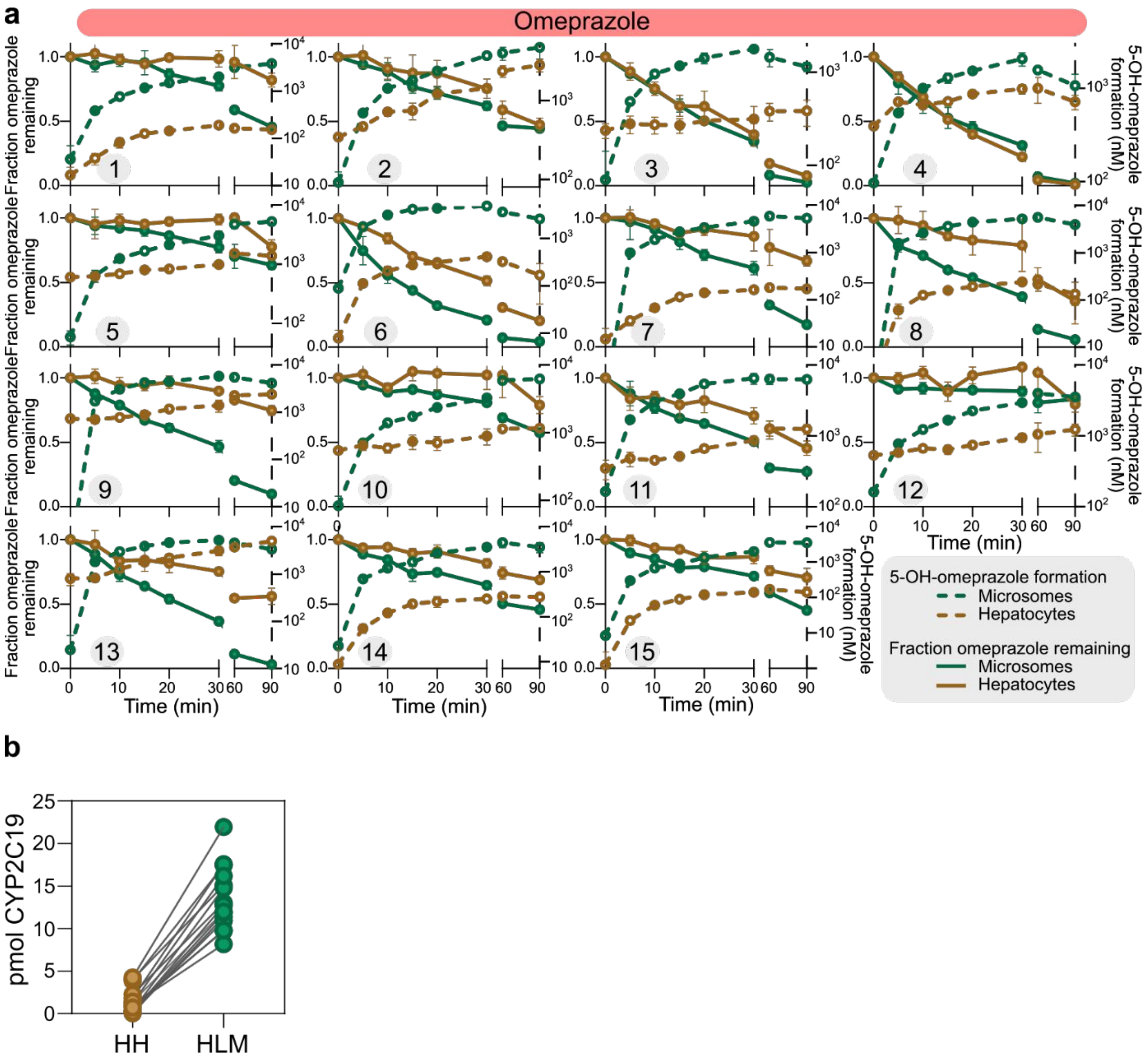

Figure S7. Metabolic clearance of omeprazole. a) Metabolic clearance of omeprazole, measured by depletion of omeprazole (fraction remaining) and 5-OH-omeprazole formation in HLM and HH from 15 matching donors. b) Amount of CYP2C19 in the incubations of the 15 donors in HLM and HH, respectively.

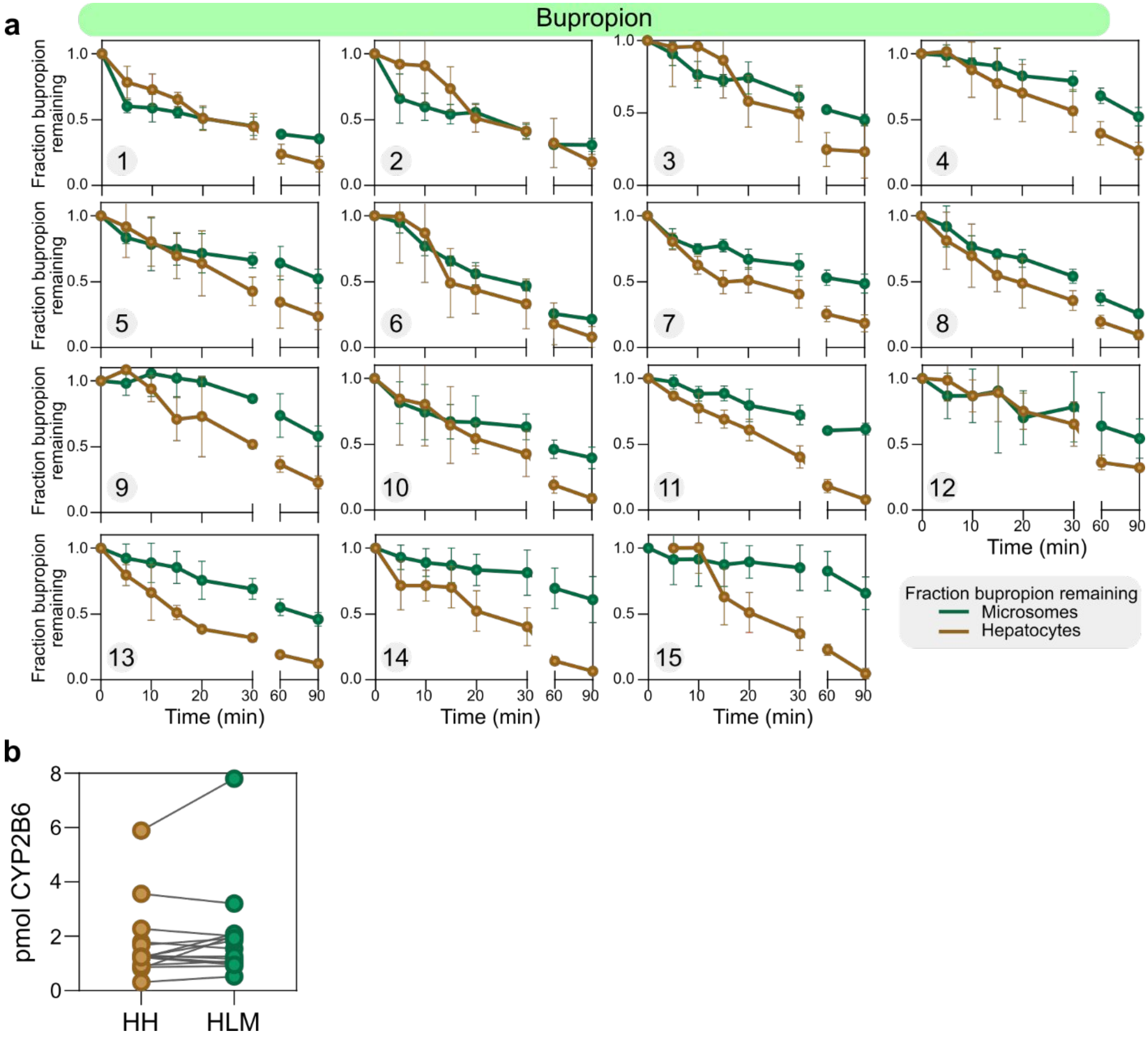

Figure S8. Metabolic clearance of bupropion. a) Metabolic clearance of bupropion, measured by depletion of bupropion (fraction remaining) in HLM and HH. b) Amount of CYP2B6 in the incubations of the 15 donors in HLM and HH, respectively.

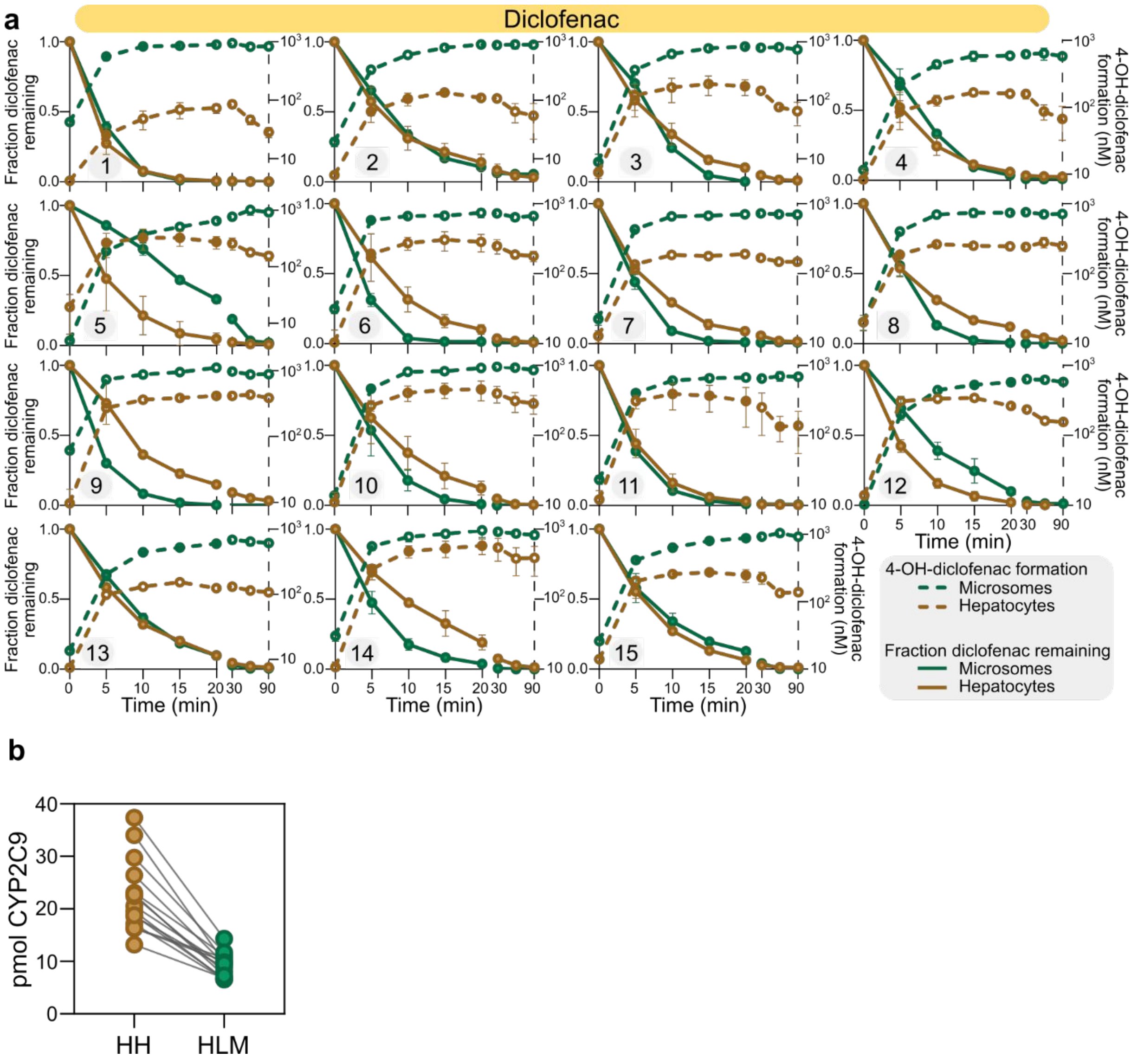

Figure S9. Metabolic clearance of diclofenac. a) Metabolic clearance of diclofenac, measured by depletion of diclofenac (fraction remaining) and 4-OH-diclofenac formation in HLM and HH from 15 matching donors. b) Amount of CYP2C9 in the incubations of the 15 donors in HLM and HH, respectively.

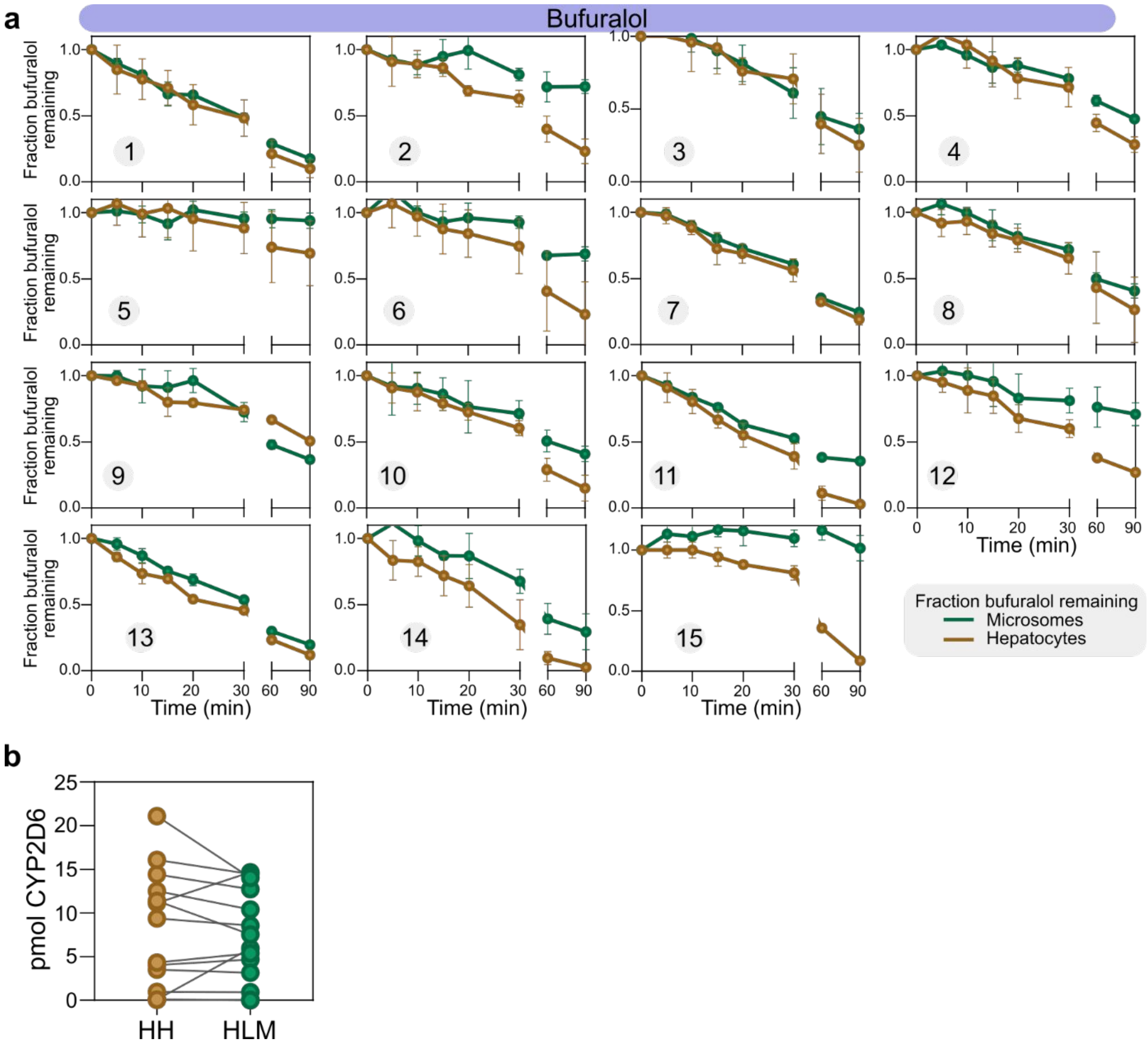

Figure S10. Metabolic clearance of bufuralol. a) Metabolic clearance of bufuralol, measured by depletion of bufuralol (fraction remaining) in HLM and HH. b) Amount of CYP2D6 in the incubations of the 15 donors in HLM and HH, respectively.

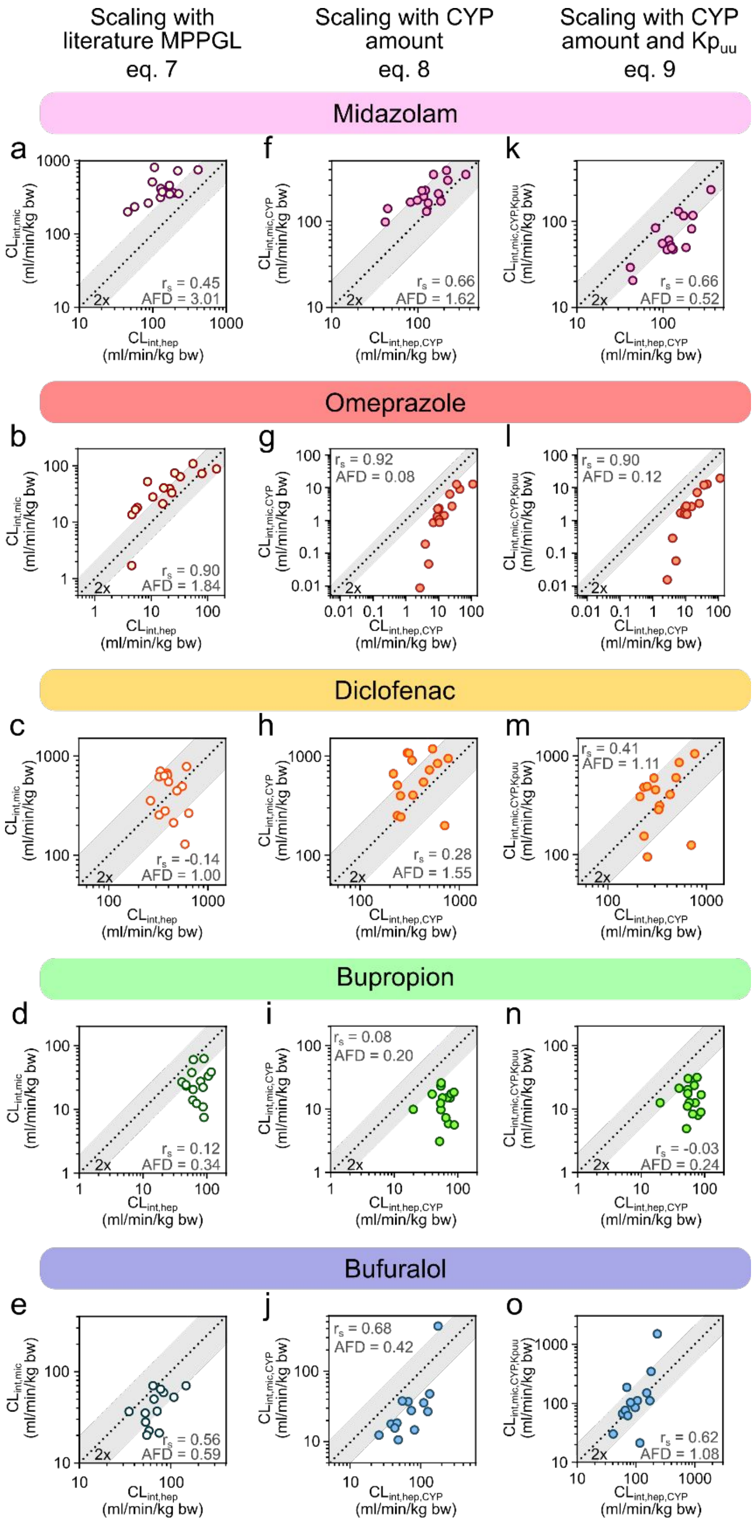

Figure S11. Adjusting $CL_{int,mic}$ and $CL_{int,hep}$ with factors influencing metabolic clearance. a-e) Unbound *in vitro* clearance was scaled to kg body weight (bw) from HH with hepatocellularity and HLM with literature MPPGL (eq.7), for five probe CYP substrates (midazolam, omeprazole, diclofenac, bupropion, and bufuralol) in 15 donor-matched HLM and HH **f-j)** Unbound *in vitro* clearance was scaled to kg bw with specific CYP amount (midazolam – CYP3A4, omeprazole – CYP2C19, diclofenac – CYP2C9, bupropion – CYP2B6, and bufuralol – CYP2D6) in HH and HLM, respectively and CYP concentration in HL (eq. 8). **k-o)** HLM clearance scaled with CYP amount was adjusted with Kp_{uu} determined in HH (eq. 9). r_s = Spearman's rank correlation coefficient; AFD = Average fold difference.

References

1. Liebermeister, W., et al., *Visual account of protein investment in cellular functions.* Proceedings of the National Academy of Sciences, 2014. **111**(23): p. 8488.
2. Palade, G.E. and P. Siekevitz, *Liver microsomes.* The Journal of Biophysical and Biochemical Cytology, 1956. **2**(2): p. 171.
3. Thul, P.J., et al., *A subcellular map of the human proteome.* Science, 2017. **356**(6340): p. eaal3321.
4. Alberts, B., et al., *Fractionation of cells*, in *Molecular Biology of the Cell. 4th edition*. 2002, Garland Science.
5. Andringa, K.K. and S.M. Bailey, *Chapter Six - Detection of Protein Thiols in Mitochondrial Oxidative Phosphorylation Complexes and Associated Proteins*, in *Methods in Enzymology*, E. Cadenas and L. Packer, Editors. 2010, Academic Press. p. 83-108.
6. Graham, J., *Preparation of Crude Subcellular Fractions by Differential Centrifugation* TheScientificWorldJournal, 2002. **2**.
7. Lodish, H., et al., *Purification of cells and their parts*, in *Molecular Cell Biology. 4th edition*. 2000, WH Freeman.
8. Achour, B., et al., *Global Proteomic Analysis of Human Liver Microsomes: Rapid Characterization and Quantification of Hepatic Drug-Metabolizing Enzymes.* Drug Metabolism and Disposition, 2017. **45**(6): p. 666.
9. Couto, N., et al., *Quantification of Proteins Involved in Drug Metabolism and Disposition in the Human Liver Using Label-Free Global Proteomics.* Molecular Pharmaceutics, 2019. **16**(2): p. 632-647.
10. Xu, M., et al., *Targeted LC-MS/MS Proteomics-Based Strategy To Characterize in Vitro Models Used in Drug Metabolism and Transport Studies.* Analytical Chemistry, 2018. **90**(20): p. 11873-11882.
11. Hamilton, R.L., et al., *A rapid calcium precipitation method of recovering large amounts of highly pure hepatocyte rough endoplasmic reticulum.* Journal of lipid research, 1999. **40**(6): p. 1140-1147.
12. Zanger, U.M. and M. Schwab, *Cytochrome P450 enzymes in drug metabolism: Regulation of gene expression, enzyme activities, and impact of genetic variation.* Pharmacology & Therapeutics, 2013. **138**(1): p. 103-141.
13. Wegler, C., et al., *Variability in Mass Spectrometry-based Quantification of Clinically Relevant Drug Transporters and Drug Metabolizing Enzymes.* Molecular Pharmaceutics, 2017. **14**(9): p. 3142-3151.
14. Wiśniewski, J.R., et al., *In-depth quantitative analysis and comparison of the human hepatocyte and hepatoma cell line HepG2 proteomes.* Journal of Proteomics, 2016. **136**: p. 234-247.
15. Weiß, F., et al., *Direct Quantification of Cytochromes P450 and Drug Transporters—A Rapid, Targeted Mass Spectrometry-Based Immunoassay Panel for Tissues and Cell Culture Lysates.* Drug Metabolism and Disposition, 2018. **46**(4): p. 387-396.
